# Circulating Platelet–Neutrophil Aggregates as Novel Biomarker for Coagulopathy Diagnosis and Disseminated Intravascular Coagulation Prediction in Sepsis

**DOI:** 10.1155/mi/5580762

**Published:** 2026-03-23

**Authors:** Tenglong Dai, Shuang Liang, Bin Li, Huiru Zhao, Yu Su, Ying Liu, Cuiying Liang, Xinyang Yue, Hao Wang, Jun Wu

**Affiliations:** ^1^ Thrombosis Research Center, Peking University Fourth School of Clinical Medicine, Beijing, China; ^2^ Thrombosis Research Center, Beijing Jishuitan Hospital, Capital Medical University, Beijing, China, ccmu.edu.cn; ^3^ Division of Critical Care Medicine, Beijing Jishuitan Hospital, Capital Medical University, Beijing, China, ccmu.edu.cn; ^4^ Department of Blood Transfusion, Beijing Jishuitan Hospital, Capital Medical University, Beijing, China, ccmu.edu.cn

**Keywords:** disseminated intravascular coagulation, platelet–leukocyte aggregates, platelet–neutrophil aggregates, sepsis-induced coagulopathy, thrombosis

## Abstract

**Background:**

Platelet–leukocyte aggregates (PLAs) drive immunothrombosis by promoting leukocyte activation and tissue factor release in sepsis. However, their utility as early predictors of sepsis‐induced coagulopathy (SIC) and disseminated intravascular coagulation (DIC) remains unestablished. This study aimed to develop and validate a PLA‐based model for SIC diagnosis and DIC prediction.

**Methods:**

A prospective prediction study was conducted with 101 sepsis patients in Beijing Jishuitan Hospital between May 2024 and May 2025. Percentage and mean fluorescence intensity (MFI) of different subtypes of PLAs were measured by flow cytometry. Receiver operating characteristic (ROC) curves and Cox regression were performed, assessing the performance of PLAs to diagnose SIC and predict DIC.

**Results:**

Platelet–neutrophil aggregate MFI (PNA‐MFI) demonstrated excellent diagnostic accuracy for SIC (*p* < 0.001, AUC = 0.789, 95% CI: 0.699–0.880, cut‐off value = 2797, sensitivity = 76.2%, specificity = 78.0%, negative predictive value (NPV) = 83.9%, and positive predictive value (PPV) = 68.8%) and superior DIC prediction (*p* < 0.001, AUC = 0.781, 95% CI: 0.680–0.883, cut‐off value = 2621, sensitivity = 83.3%, specificity = 64.9%, NPV = 92.6%, and PPV = 42.6%). Kaplan–Meier analysis showed that high PNA‐MFI patients (PNA‐MFI ≥ 2621) were more likely to develop DIC (log‐rank *p*  < 0.001, hazard ratio: 6.80 [high PNA‐MFI/low PNA‐MFI], and 95% CI:2.97–15.61).

**Conclusion:**

PNA‐MFI is a promising biomarker for SIC diagnosis and DIC prediction. While internally validated, external validation is essential prior to clinical implementation, and further multicenter studies are warranted to definitively establish its clinical value.

## 1. Introduction

Sepsis‐associated disseminated intravascular coagulation (DIC) is a progressive and severe condition characterized by systemic coagulation activation, manifesting as intravascular fibrin formation, microangiopathic thrombosis, and consumptive coagulopathy (depletion of coagulation factors and platelets). As the most prevalent life‐threatening complication of sepsis [1], DIC carries >40% mortality upon development [2, 3]. This complication originates from sepsis‐induced coagulopathy (SIC), defined as an early phase of DIC [4]. During SIC, septic patients develop a prothrombotic state mediated by the extrinsic pathway, cytokine‐induced amplification of coagulation, suppression of anticoagulant pathways, and impaired fibrinolysis [5]. Despite standardized SIC scoring for early DIC detection [6, 7], the absence of validated biomarkers for SIC diagnosis and DIC risk stratification remains a critical unmet need [8].

Immunothrombosis, an interplay between a broad inflammatory response and coagulation activation is a distinguishing feature of sepsis [9]. Platelets, leukocytes, and the interaction between platelets and leukocytes are pivotal in this process [10–12]. Activated platelets binding to different subtypes of leukocytes form mixed aggregates called platelet–leukocyte aggregates (PLAs) [13], including platelet–neutrophil aggregates (PNAs), platelet–eosinophil aggregates (PEAs), platelet–monocyte aggregates (PMAs), and platelet–lymphocyte aggregates (PLyAs). PLAs are considered a reliable marker of a prothrombotic state and are associated with several thrombosis diseases [14, 15]. The binding of platelet P‐selectin to P‐selectin glycoprotein ligand‐1 (PSGL‐1) on leukocytes is a crucial molecular mechanism in PLA cellular interaction and also induces inflammatory reactions, thereby contributing to the thrombotic progression [16, 17]. For instance, the binding of platelet P‐selectin to PSGL‐1 on neutrophils triggers NETosis and promotes thrombosis during sepsis in mice [18, 19]. In sepsis, neutrophil extracellular traps (NETs) stimulate platelets and trigger the coagulation cascade [20]. Pathogen‐associated molecular patterns (PAMPs) from microorganisms stimulate TF expression in monocytes, which is a key mechanism initiating coagulation cascade activation [21, 22]. For example, lipopolysaccharide (LPS) endotoxemia increases PMA, TF expression, and platelet activation [23]. Thus, PLAs provide a novel link between inflammation and thrombosis, two central processes in SIC.

We hypothesized that PLAs, by bridging immunothrombosis through mechanisms described above, could provide critical diagnostic and prognostic insights into SIC pathogenesis, potentially offering a novel stratification tool to guide targeted interventions in SIC patients. To test this hypothesis, we conducted a prospective prediction study evaluating PLA subtypes as dual‐purpose biomarkers for SIC diagnosis and DIC risk prediction.

## 2. Materials and Methods

### 2.1. Patients

A prospective prediction study was conducted on sepsis patients admitted to the ICU at Beijing Jishuitan Hospital from May 2024 to May 2025. The samples from septic patients with coagulopathy were collected within 24 h after diagnosis, and matched septic non‐SIC patients were selected for comparison, with samples collected within the same time frame. Sepsis was defined as a complete sequential organ failure assessment (SOFA) score of ≥2 points due to infection [24]. The DIC and SIC scores were computed based on criteria from the International Society of Thrombosis and Haemostasis (ISTH) [25, 26]. The DIC score was based on platelet count, prothrombin time (PT), D‐dimer levels (<5 mg/L FEU = 1 point, 5–10 mg/L FEU = 2 points, and ≥10 mg/L FEU = 3 points), and fibrinogen (FIB) levels. A DIC score of ≥5 was considered indicative of overt DIC. Similarly, the SIC score was calculated using platelet count, international normalized ratio (INR), and SOFA score. An SIC score of ≥4 was used to define SIC. The exclusion criteria were as follows: (1) no insufficient information to meet sepsis diagnostic criteria, (2) under 18 years old, (3) pregnant, and (4) receiving antiplatelet therapy.

The study received approval from the Ethics Committee of Beijing Jishuitan Hospital. Patients and their immediate family members provided written informed consent. The supervising clinician ensured that patients were fully informed and understood the study before signing the consent form.

### 2.2. Clinical and Laboratory Evaluation

Clinical history evaluation, laboratory tests, and plasma PLA analysis were carried out, including age, sex, blood routine examination, coagulation parameters, and SOFA score. Blood routine examination was analyzed using a Sysmex XT4000i analyzer, including red blood cell (RBC), white blood cell (WBC), and platelet counts. Coagulation parameters were analyzed by Sysmex CS2100i analyzer, including activated partial thromboplastin time (APTT), PT, INR, and FIB and D‐dimer measurements. PLAs were measured by flow cytometry (BD FACS Canto Ⅱ flow cytometer, USA). The provided antibodies are mouse antihuman monoclonal antibodies (BioLegend, San Diego, CA). PLAs were categorized into three groups: PLyAs, platelet–granulocyte aggregates (PGAs), and PMAs. PLyAs were identified using PerCP/Cyanine5.5 antihuman CD4, APC antihuman CD19, FITC antihuman CD3, APC/Cyanine7 antihuman CD8, and PE antihuman CD61 antibodies. PGAs were identified with APC/Cyanine7 antihuman CD11b, PE/Cyanine7 antihuman CD15, PerCP/Cyanine5.5 antihuman CD16, and PE antihuman CD61 antibodies. PMAs were identified using APC antihuman CD14, PerCP/Cyanine5.5 antihuman CD16, and PE antihuman CD61 antibodies. After incubation with antibodies in the dark, samples were simultaneously fixed, and erythrocytes were lysed using a commercial lysing solution containing 3% paraformaldehyde (Becton Dickinson, USA) [27]. This critical step stabilizes platelet surface markers and halts cellular activation, thereby preserving the in vivo state of the PLAs for analysis [28–30]. Fixed samples were then washed via centrifugation to remove the lysing solution before flow cytometric analysis [31]. Data were collected using the BD FACS Canto II flow cytometer and processed with FlowJo Software v10.10. The specific gating strategy is shown in Supporting Information 1: Figure S1, Supporting Information 2: Figure S2, and Supporting Information 3: Figure S3, along with their corresponding legends. In the granulocyte gate, neutrophils are defined as CD11b^+^CD15^+^CD16^+^, while eosinophils are CD11b^+^CD15^+^CD16^−^. In the lymphocyte gate, T‐lymphocytes are defined as CD3^+^, CD4^+^T‐lymphocytes as CD3^+^CD4^+^, CD8^+^T‐lymphocyte as CD3^+^CD8^+^, and B‐lymphocyte as CD3^−^CD19^−^. In the monocyte gate, monocytes (All) represent CD14^+^, and classical monocytes are defined as CD14^++^CD16^−^. PLAs were defined as events positive for both leukocyte markers and the platelet marker CD61 (glycoprotein IIIa). In this study, the level of percentage and mean fluorescence intensity (MFI) were analyzed in different PLAs groups as PLA indicators.

### 2.3. Statistical Analysis

This study evaluated two distinct outcomes: SIC as a diagnostic outcome, assessed at enrollment using ISTH criteria; DIC as a prognostic outcome, defined as overt DIC development within 75 days postsepsis diagnosis. Statistical differences between cohorts were analyzed using the Mann–Whitney *U* test for continuous variables and Pearson’s χ^2^ test for categorical variables. The diagnostic/predictive performance of each indicator was quantified by the area under the receiver operating characteristic (ROC) curve (AUC). The significance of an individual AUC was tested against the null hypothesis of no discrimination (AUC = 0.5), with results reported as *p* ≤ 0.05. To compare performance between any two indicators for the same endpoint, pairwise comparisons of their AUCs were conducted using DeLong‘s test for correlated ROC curves. In the main text, significant pairwise findings are reported as *p* = 0.05, by DeLong‘s test. For time‐to‐event analysis of the prognostic outcome (DIC), the observation period was strictly limited to 75 days following sepsis diagnosis. Time zero was defined as the date of sepsis diagnosis, with the endpoint for the prognostic outcome being the development of overt DIC within this timeframe. Kaplan–Meier curves with log‐rank tests were employed. Statistical significance was defined as *p* ≤ 0.05. All calculations were performed using GraphPad Prism 9 and SPSS 24 statistical software (SPSS Inc., USA), along with R software (version 4.2.1).

## 3. Results

### 3.1. Basic Characteristics

This study included a total of 101 patients diagnosed with sepsis, with 42 of them having coagulopathy and 59 without coagulopathy. The basic characteristics of all the patient are shown in Table 1. No differences were identified between the groups in gender, age, site of infection, or medical history, except for a higher incidence of fungal infection in the SIC group. It is noteworthy that patients with SIC exhibited decreased platelet counts. Moreover, the SIC group demonstrated substantially prolonged PT, lower PT activity% (PA%), reduced FIB levels, elevated INR, and higher SOFA scores compared to the non‐SIC group (Table 1).

**Table 1:** Demographics and biomarkers in sepsis patients with and without SIC.^a^

Variables	Non‐SIC *N* = 59	SIC *N* = 42	*p*‐Value
Characteristics			
Male sex (*n*, %)	37 (64)	30 (71)	0.423
Age, years	64.00 (56.00, 78.00)	72.00 (62.00, 77.00)	0.111
Mortality day 90 (*n*, %)	11 (19)	12 (29)	0.260
Site of infection (*n*, %)			0.917
Bloodstream infection (Gram‐positive cocci)	8 (14)	4 (10)	
Bloodstream infection (Gram‐negative bacilli)	6 (10)	3 (7)	
Multiple infections	11 (19)	10 (24)	
Pulmonary infection	29 (50)	22 (52)	
Unknown	4 (7)	3 (7)	
Virus (*n*, %)	10 (17)	7 (17)	0.940
Fungus (*n*, %)	12 (21)	19 (45)	0.009
Medical conditions			
Hypertension (*n*, %)	31 (53)	22 (52)	0.916
Diabetes mellitus (*n*, %)	16 (28)	14 (33)	0.536
Coronary heart disease (*n*, %)	15 (26)	10 (24)	0.815
Cerebral infarction (*n*, %)	14 (24)	5 (12)	0.124
Cancer (*n*, %)	11 (19)	4 (10)	0.192
Clinical characteristics			
RBC (10^12^/L)	2.89 (2.55, 3.24)	2.69 (2.49, 3.31)	0.313
WBC (10^9^/L)	11.50 (8.41, 14.75)	10.09 (6.94, 14.23)	0.128
PLT (10^9^/L)	206.50 (149.00, 279.80)	78 (60.50, 96.00)	<0.001
PT (s)	14.00 (13.02, 14.80)	15.20 (12.86, 16.75)	0.002
PA (%)	77.30 (67.80, 85.00)	70.30 (54.40, 85.60)	0.035
INR	1.14 (1.08, 1.22)	1.27 (1.10, 1.42)	0.005
APTT (s)	33.30 (28.30, 41.00)	33.97 (29.35, 43.79)	0.401
FIB (mg/dL)	421.0 (300.4, 566.0)	343.80 (255.3, 477.5)	0.036
D‐dimer (mg/L FEU)	4.06 (2.35, 7.56)	4.24 (2.07, 9.98)	0.986
TT (s)	15.65 (14.65, 17.06)	16.05 (14.83, 18.88)	0.108
SOFA score	3.00 (2.00, 5.00)	6.00 (4.00, 8.00)	<0.001

*Note:* PA, prothrombin time activity.

Abbreviations: APTT, activated partial thromboplastin time; DIC, disseminated intravascular coagulation; INR, international normalized ratio; PT, prothrombin time; SIC, sepsis‐induced coagulopathy; SOFA, sequential organ failure assessment; TT, thrombin time.

^a^Values are expressed as median (25th and 75th percentiles) or number (percentage). *p* < 0.05 is considered statistically significant.

### 3.2. Comparison of PLA Indicators Between SIC and Non‐SIC Patients

Flow cytometry was used to detect PLA indicators in peripheral whole blood samples from the 71 participants. Compared to non‐SIC patients, the levels of PNA % (4.22% vs. 5.26%, 95% CI = −2.35–−0.22, and *p* = 0.021, Figure 1A), PEA % (3.84% vs. 6.30%, 95% CI = −4.22–−0.71, and *p* = 0.007, Figure 1B), PLyA (T‐lymphocyte) % (4.76% vs. 7.13%, 95% CI = −3.90–−0.72, and *p* = 0.007, Figure 1E), PLyA (CD4^+^T‐lymphocyte) % (3.71% vs. 6.13%, 95% CI = −3.90–−0.85, and *p* = 0.001, Figure 1F), PLyA (CD8^+^T‐lymphocyte) % (4.89% vs. 8.12%, 95% CI = −4.18–−0.47, and *p* = 0.016, Figure 1G), and PLyA (B‐lymphocyte) % (5.61% vs. 8.52%, 95% CI = −5.15–−0.85, and *p* = 0.005, Figure 1H) were lower compared to non‐SIC patients. In the level of MFI, SIC patients showed elevated PNA‐MFI (3709 vs. 2119, 95% CI = 825–1762, and *p*  < 0.001, Figure 2A), PMA (All) MFI (6207 vs. 3793, 95% CI = 1121–3145, and *p*  < 0.001, Figure 2C), PMA (classical‐monocyte) MFI (3411 vs. 2140, 95% CI = 785–1880, and *p*  < 0.001, Figure 2D), PLyA (T‐lymphocyte) MFI (3708 vs. 2100, 95% CI = 758–1807, and *p*  < 0.001, Figure 2E), PLyA (CD4^+^T‐lymphocyte) MFI (3087 vs. 1726, 95% CI = 423–1578, and *p*  < 0.001, Figure 2F), PLyA (CD8^+^T‐lymphocyte) MFI (3700 vs. 2133, 95% CI = 583–1817, and *p*  < 0.001, Figure 2G), and PLyA (B‐lymphocyte) MFI (3642 vs. 2081, 95% CI = 859–2019, and *p*  < 0.001, Figure 2H). The interaction between platelets and different leukocyte subtypes is stimulated. However, no differences were observed in PMA (all) % (4.23% vs. 5.67%, 95% CI = −2.89–0.21, and *p* = 0.086, Figure 1C), PMA (classical‐monocyte) % (4.20% vs. 5.30%, 95% CI = −2.83–0.23, and *p* = 0.098, Figure 1D), and PEA‐MFI (2993 vs. 2141, 95% CI = −120–1147, and *p* = 0.102, Figure 2B) between SIC and non‐SIC patients. *p*‐Values for 16 PLA indicators in SIC vs. non‐SIC comparisons were adjusted by false discovery rate (FDR) correction and Bonferroni correction. Adjusted *p*‐values are presented in Supporting Information 4: Table S1.

Figure 1Distribution difference of the percentage level of PLAs in non‐SIC and SIC among septic patients. The level of percentage of PLAs was compared among patients without SIC (non‐SIC, *n* = 59; blue bars) and patients with SIC (*n* = 42; red bars). The variables were compared using a Mann–Whitney test. Graph shows comparison of percentage of (A) platelet–neutrophil aggregates, (B) platelet–eosinophil aggregates, (C) platelet–monocyte aggregates, (D) platelet classical–monocyte aggregates, (E) platelet T–lymphocyte aggregates, (F) platelet CD4^+^T–lymphocyte aggregates, (G) platelet CD8^+^T–lymphocyte aggregates, and (H) platelet B–lymphocyte aggregates between non‐SIC group and SIC group.  ^∗^ *p* < 0.05;  ^∗∗^ *p* < 0.01;  ^∗∗∗^ *p* < 0.001, compared with non‐SIC. SIC, sepsis‐induced coagulopathy; PNAs, platelet–neutrophil aggregates; PEAs, platelet–eosinophil aggregates; PMAs, platelet–monocyte aggregates; PLyAs, platelet–lymphocyte aggregates.

**Figure:**
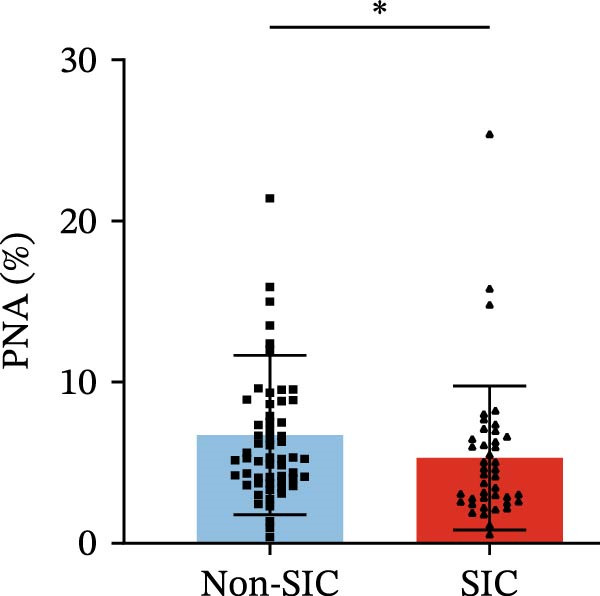
(A)

**Figure:**
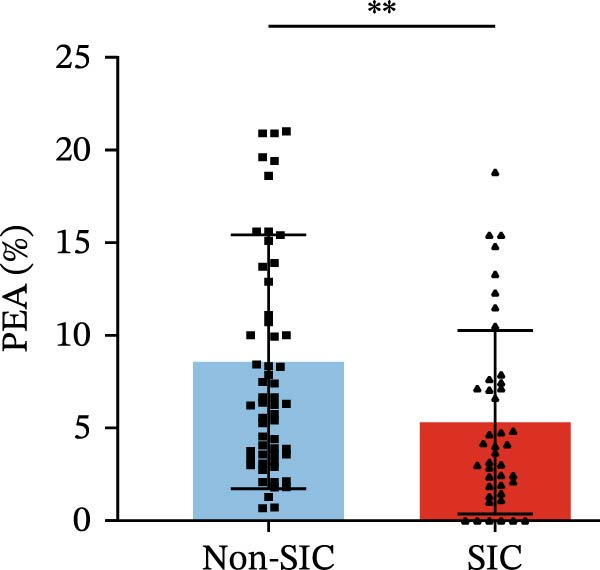
(B)

**Figure:**
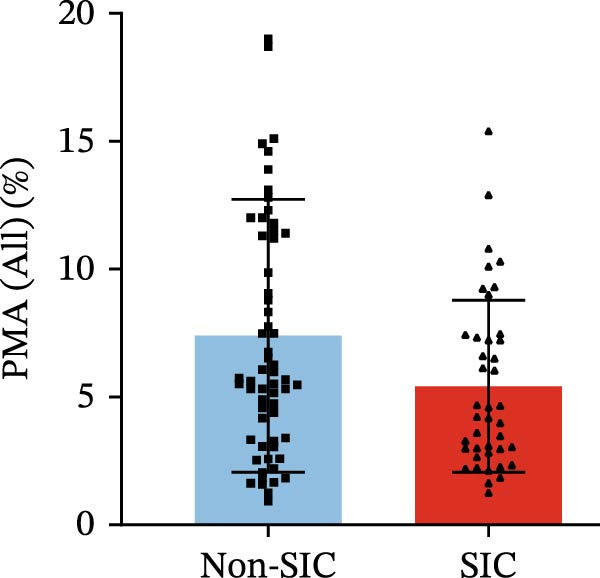
(C)

**Figure:**
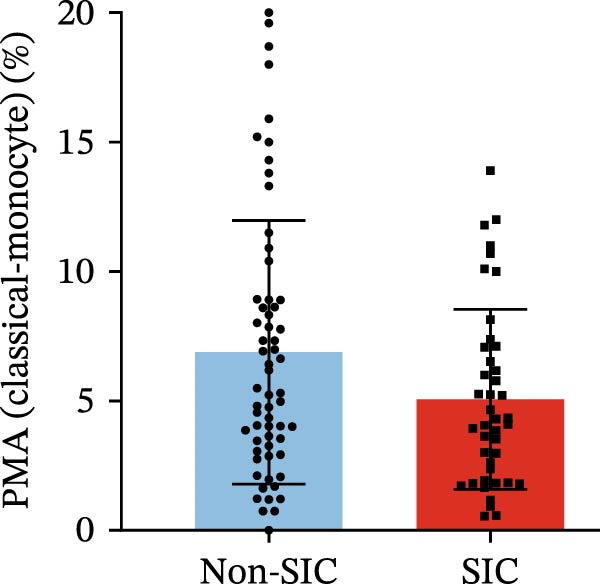
(D)

**Figure:**
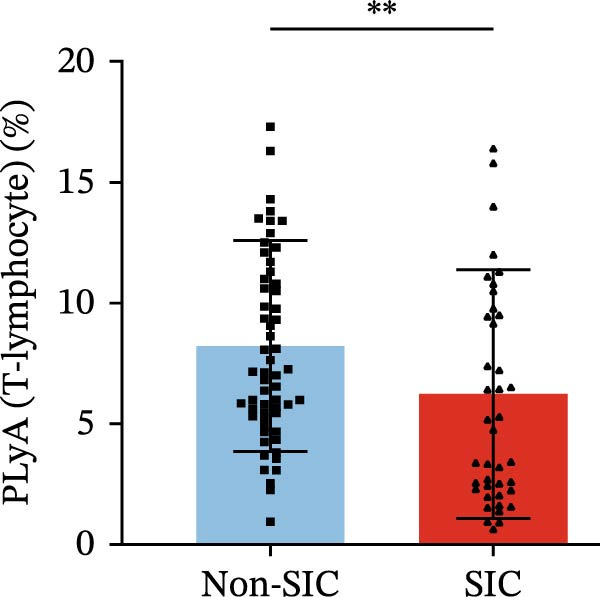
(E)

**Figure:**
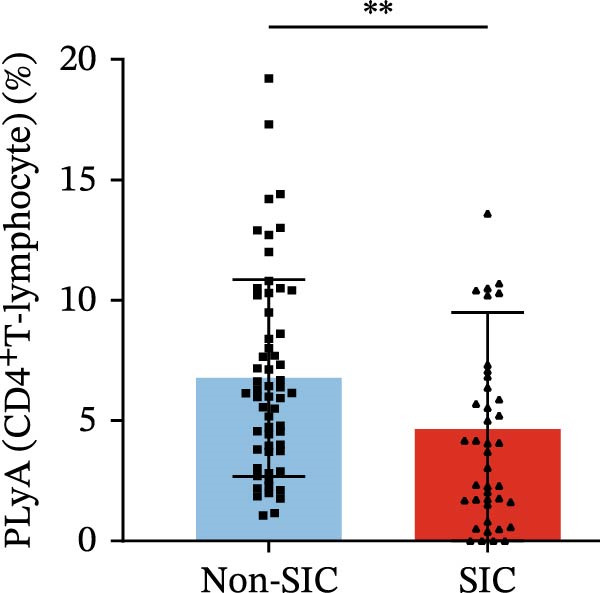
(F)

**Figure:**
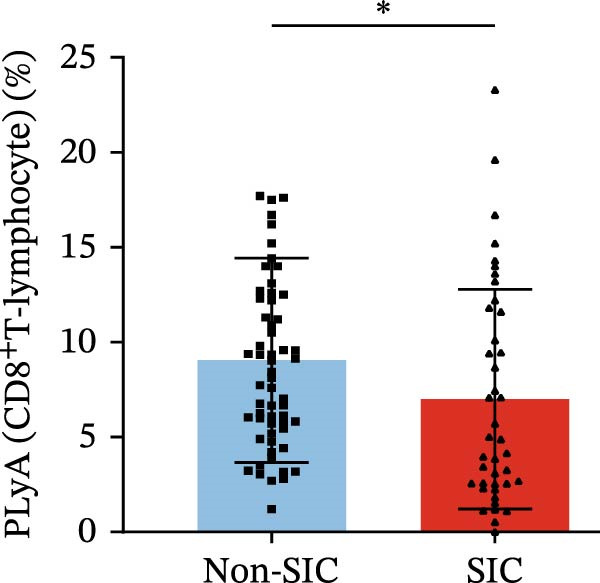
(G)

**Figure:**
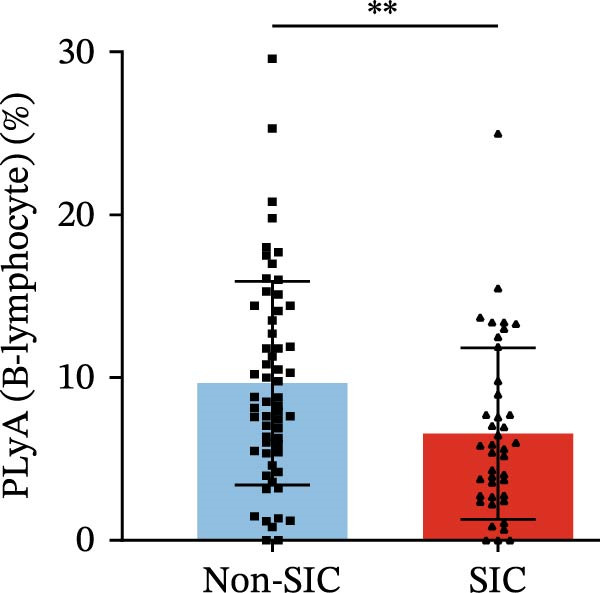
(H)

Figure 2Distribution difference of the MFI of PLAs in non‐SIC and SIC among septic patients. Graph shows comparison of (A) PNA‐MFI, (B) PEA‐MFI, (C) PMA (All)‐MFI, (D) PMA (classical‐monocyte)‐MFI, (E) PLyA (T‐lymphocyte)‐MFI, (F) PLyA (CD4^+^T‐lymphocyte)‐MFI, (G) PLyA (CD8^+^T‐lymphocyte)‐MFI, and (H) PLyA (B‐lymphocyte)‐MFI between non‐SIC group and SIC group.  ^∗^ *p* < 0.05;  ^∗∗^ *p* < 0.01;  ^∗∗∗^ *p* < 0.001, compared with non‐SIC. SIC, sepsis‐induced coagulopathy; MFI, mean fluorescence intensity; PNA‐MFI, platelet–neutrophil aggregate mean fluorescence intensity; PEA‐MFI, platelet–eosinophil aggregate mean fluorescence intensity; PMA (all)‐MFI, platelet–monocyte aggregate mean fluorescence intensity; PMA (classical‐monocyte)‐MFI, platelet classical–monocyte aggregate mean fluorescence intensity; PLyA (T‐lymphocyte)‐MFI, platelet T–lymphocyte aggregate mean fluorescence intensity; PLyA (CD4^+^T‐lymphocyte)‐MFI, platelet CD4^+^T–lymphocyte aggregate mean fluorescence intensity; PLyA (CD8^+^T‐lymphocyte)‐MFI, platelet CD8^+^T–lymphocyte aggregate mean fluorescence intensity; PLyA (B‐lymphocyte)‐MFI, platelet B–lymphocyte aggregate mean fluorescence intensity.

**Figure:**
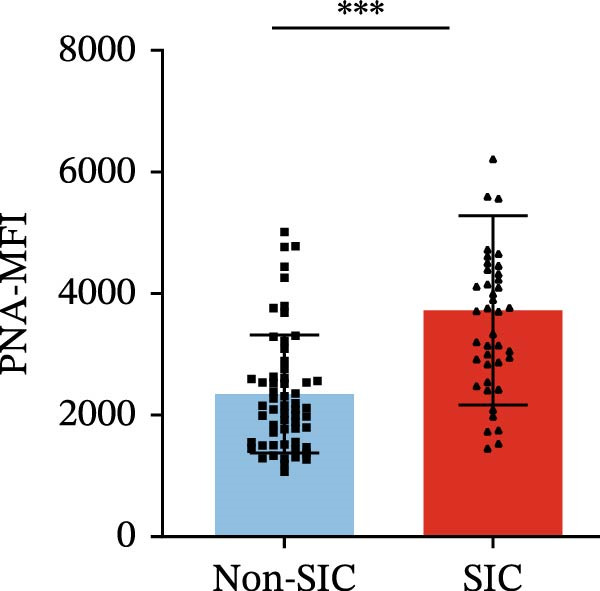
(A)

**Figure:**
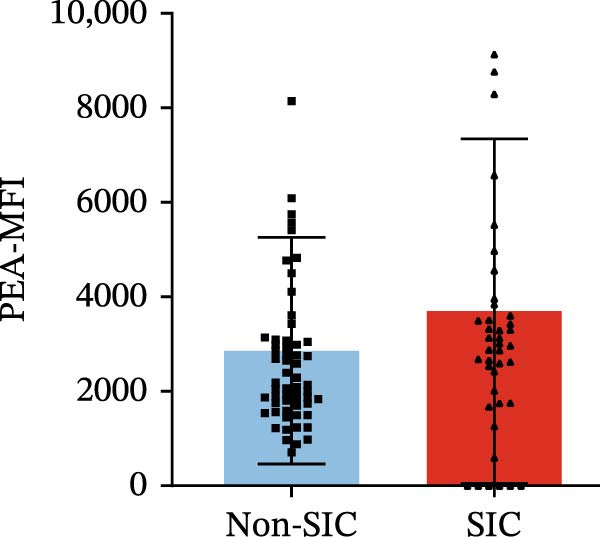
(B)

**Figure:**
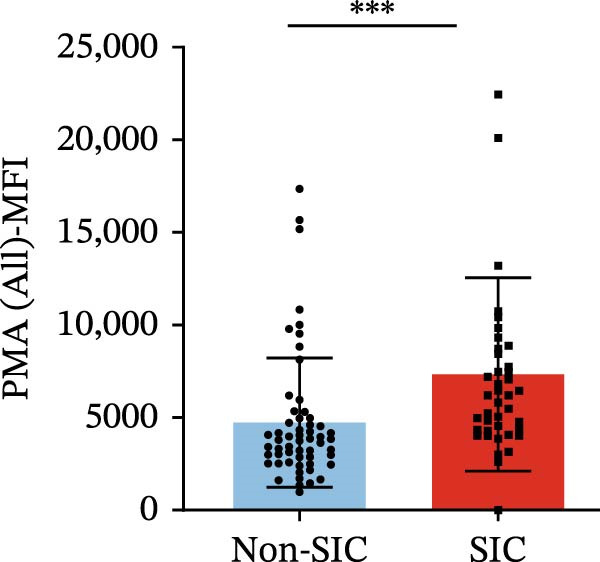
(C)

**Figure:**
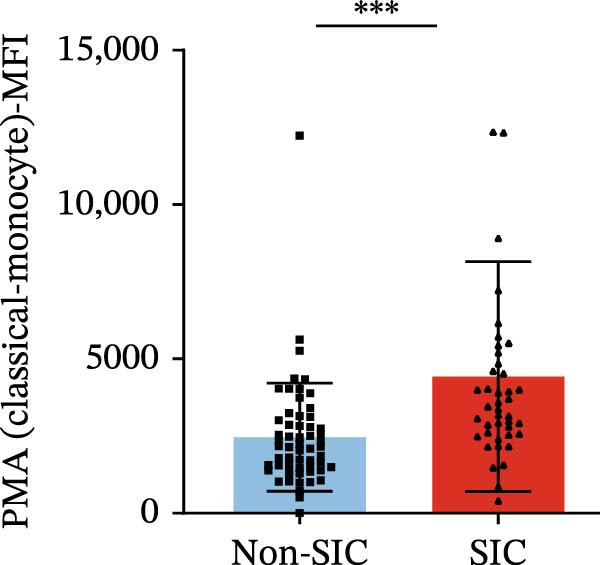
(D)

**Figure:**
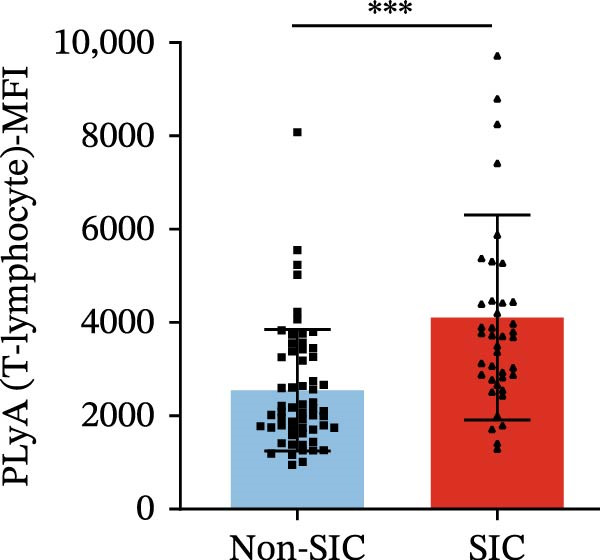
(E)

**Figure:**
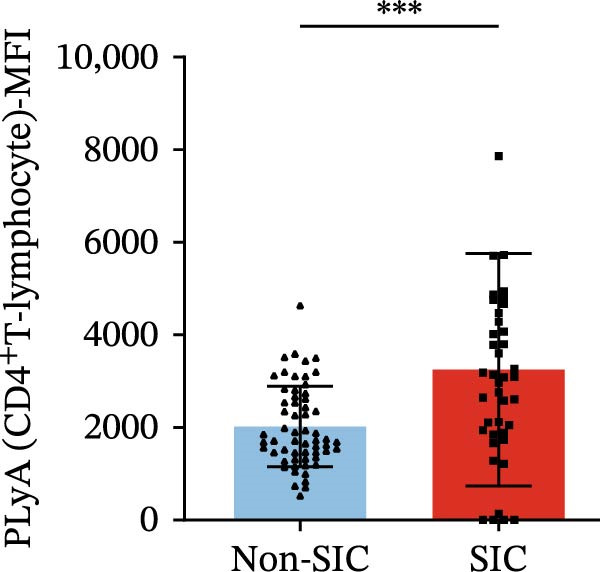
(F)

**Figure:**
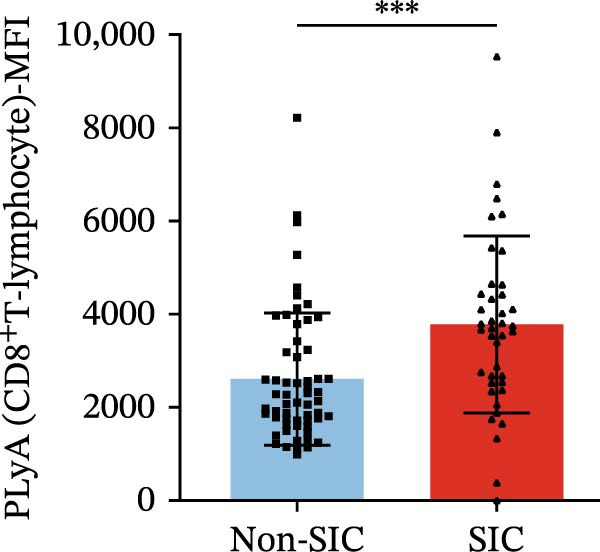
(G)

**Figure:**
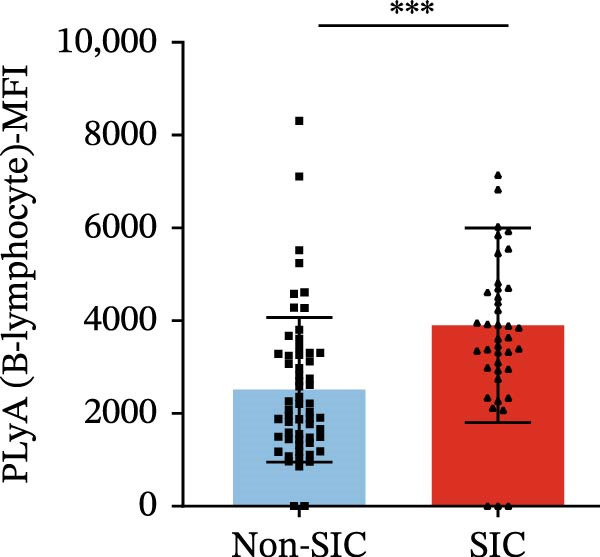
(H)

### 3.3. Diagnostic Value of PLA Indicators in SIC

We conducted correlation analysis to identify PLA indicators that are strongly associated with SIC. Subsequently, we conducted a ROC analysis to assess whether PLA indicators can be used to distinguish between sepsis with coagulopathy and sepsis without coagulopathy. The results, including the AUC, sensitivity and specificity values, cut‐off values, positive predictive value (PPV), negative predictive value (NPV), and significance, are presented in Table 2. Upon comparing the ROC for various indicators, we have identified PNA%, PEA%, PLyA (T‐lymphocyte) %, PLyA (CD4^+^T‐lymphocytes) %, PLyA (CD8^+^T‐lymphocytes) %, PLyA (B‐lymphocytes) %, PNA‐MFI, PMA (all)‐MFI, PMA (classical‐monocyte)‐MFI, PLyA (T‐lymphocyte)‐MFI, PLyA (CD4^+^T‐lymphocytes)‐MFI, PLyA (CD8^+^T‐lymphocyte)‐MFI, and PLyA (B‐lymphocyte)‐MFI that have shown statistical significance (Table 2). These molecules are capable of differentiating between patients with coagulopathy and those without. PNA‐MFI demonstrated significant diagnostic utility for SIC, with an AUC of 0.789. To assess its performance relative to other cellular markers, pairwise comparisons were conducted using DeLong’s test. The results confirmed that the AUC of PNA‐MFI was not statistically superior to that of most other PLA indicators (all *p*  > 0.05) (Table 3). Despite the lack of statistical superiority in pairwise comparisons, its robust and standalone diagnostic performance solidifies PNA‐MFI as a substantively meaningful marker for SIC.

**Table 2:** Diagnostic test characteristics of PLA indicators for sepsis‐induced coagulopathy.

PLA indicators	AUC (95% CI)	*p*‐Value	Cut‐off value^a^	Sensitivity (%)	Specificity (%)	NPV (%)	PPV (%)
PNA%	0.635 (0.523–0.746)	0.021	3.08 (%)	40.5	88.1	78.0	58.3
PEA%	0.658 (0.550–0.766)	0.007	3.17 (%)	47.6	80.0	73.3	57.1
PLyA (T‐lymphocytes) %	0.659 (0.542–0.775)	0.007	3.50 (%)	48.8	91.5	73.6	78.6
PLyA (CD4^+^T‐lymphocytes) %	0.686 (0.549–0.818)	0.002	4.31 (%)	61.0	69.5	74.1	55.3
PLyA (CD8^+^T‐lymphocyte) %	0.641 (0.523–0.760)	0.017	2.93 (%)	34.2	94.9	68.3	82.4
PLyA (B‐lymphocyte) %	0.663 (0.550–0.771)	0.006	6.01 (%)	58.5	71.2	72.4	57.1
PNA‐MFI	0.789 (0.699–0.880)	<0.001	2797	76.2	78.0	83.9	68.8
PMA (all)‐MFI	0.744 (0.649–0.844)	<0.001	4969	63.4	77.2	77.6	62.5
PMA (classical‐monocyte)‐MFI	0.754 (0.657–0.851)	<0.001	2140	90.5	50.0	84.7	64.4
PLyA (T‐lymphocyte)‐MFI	0.762 (0.667–0.857)	<0.001	2358	87.8	59.3	84.8	65.7
PLyA (CD4^+^T‐lymphocytes)‐MFI	0.703 (0.590–0.817)	<0.001	1731	80.5	50.9	72.9	61.4
PLyA (CD8^+^T‐lymphocyte)‐MFI	0.720 (0.617–0.825)	<0.001	2338	82.9	57.6	79.1	63.8
PLyA (B‐lymphocyte)‐MFI	0.758 (0.575–0.832)	<0.001	2264	87.8	55.9	83.3	65.2

*Note:* PLyA (B‐lymphocyte) %, percentage of platelet B–lymphocyte aggregates; PLyA (B‐lymphocyte)‐MFI, platelet B–lymphocyte aggregate mean fluorescence intensity; PLyA (CD4^+^T‐lymphocyte) %, percentage of platelet CD4^+^T–lymphocyte aggregates; PLyA (CD4^+^T‐lymphocyte)‐MFI, platelet CD4^+^T–lymphocyte aggregate mean fluorescence intensity; PLyA (CD8^+^T‐lymphocyte) %, percentage of platelet CD8^+^T–lymphocyte aggregates; PLyA (CD8^+^T‐lymphocyte)‐MFI, platelet CD8^+^T–lymphocyte aggregate mean fluorescence intensity; PLyA (T‐lymphocyte) %, percentage of platelet T–lymphocyte aggregates; PLyA (T‐lymphocyte)‐MFI, platelet T–lymphocyte aggregate mean fluorescence intensity; PMA (all)‐MFI, platelet–monocyte aggregate mean fluorescence intensity; PMA (classical‐monocyte)‐MFI, platelet classical–monocyte aggregate mean fluorescence intensity.

Abbreviations: AUC, area under the curve; MFI, mean fluorescence intensity; PEA%, percentage of platelet–eosinophil aggregates; PNA%, percentage of platelet–neutrophil aggregates; PNA‐MFI, platelet–neutrophil aggregate mean fluorescence intensity.

^a^All cut‐off values were determined by the Youden index.

**Table 3:** Pairwise comparisons of AUCs between PNA‐MFI and other indicators for diagnosing SIC (DeLong‘s test).

Comparison indicators (vs. PNA‐MFI)	*Z* statistic	*p*‐Value	95% CI for AUC difference
PNA%	−2.025	0.043	(−0.307, −0.005)
PEA%	−1.997	0.046	(−0.265, −0.003)
PLyA (T‐lymphocytes) %	−1.577	0.115	(−0.290, −0.031)
PLyA (CD4^+^T‐lymphocytes) %	−1.189	0.234	(−0.260, 0.064)
PLyA (CD8^+^T‐lymphocyte) %	−1.691	0.091	(−0.314, 0.023)
PLyA (B‐lymphocyte) %	−1.517	0.129	(−0.288, 0.037)
PMA (all)‐MFI	−0.582	0.561	(−0.159, 0.086)
PMA (classical‐monocyte)‐MFI	−0.573	0.567	(−0.157, 0.086)
PLyA (T‐lymphocyte)‐MFI	−0.401	0.689	(−0.151, 0.100)
PLyA (CD4^+^T‐lymphocytes)‐MFI	−1.132	0.258	(−0.230, 0.061)
PLyA (CD8^+^T‐lymphocyte)‐MFI	−0.860	0.390	(−0.218, 0.085)
PLyA (B‐lymphocyte)‐MFI	−0.538	0.591	(−0.147, 0.084)
PT	−2.050	0.040	(−0.259, −0.006)
PA (%)	−2.46	0.014	(−0.317, −0.036)
INR	−2.073	0.038	(−0.238, −0.007)
FIB	−2.076	0.038	(−0.319, −0.009)

*Note: p*‐Value ≤ 0.05 was considered significant. PLyA (B‐lymphocyte) %, percentage of platelet B–lymphocyte aggregates; PLyA (B‐lymphocyte)‐MFI, platelet B–lymphocyte aggregate mean fluorescence intensity; PLyA (CD4^+^T‐lymphocyte) %, percentage of platelet CD4^+^T–lymphocyte aggregates; PLyA (CD4^+^T‐lymphocyte)‐MFI, platelet CD4^+^T–lymphocyte aggregate mean fluorescence intensity; PLyA (CD8^+^T‐lymphocyte) %, percentage of platelet CD8^+^T–lymphocyte aggregates; PLyA (CD8^+^T‐lymphocyte)‐MFI, platelet CD8^+^T–lymphocyte aggregate mean fluorescence intensity; PLyA (T‐lymphocyte) %, percentage of platelet T–lymphocyte aggregates; PLyA (T‐lymphocyte)‐MFI, platelet T–lymphocyte aggregate mean fluorescence intensity; PMA (all) %, percentage of platelet–monocyte aggregates; PMA (all)‐MFI, platelet–monocyte aggregate mean fluorescence intensity; PMA (classical‐monocyte) %, percentage of platelet classical–monocyte aggregates; PMA (classical‐monocyte)‐MFI, platelet classical–monocyte aggregate mean fluorescence intensity.

Abbreviations: CI, confidence interval; INR, international normalized ratio; MFI, mean fluorescence intensity; PA, prothrombin time activity; PEA%, percentage of platelet–eosinophil aggregates; PEA‐MFI, platelet–eosinophil aggregate mean fluorescence intensity; PNA%, percentage of platelet–neutrophil aggregates; PNA‐MFI, platelet–neutrophil aggregate mean fluorescence intensity; PT, prothrombin time.

### 3.4. Comparative Diagnostic Performance of PNA‐MFI and Traditional Markers in SIC

Our experimental results demonstrated that PNA‐MFI exhibited the optimal diagnostic efficacy for SIC. To further investigate the clinical utility of PNA‐MFI in SIC diagnosis, we compared PNA‐MFI with conventional coagulation indicators, focusing on the AUC and *p*‐value for each indicator (Tables 3 and 4). After analysis, we found that PNA‐MFI significantly outperformed key conventional coagulation parameters—including PT, PA (%), INR, and FIB (all *p*  < 0.05, by DeLong‘s test).

**Table 4:** Diagnostic test characteristics of variables for SIC.

Variables	AUC (95% CI)	*p*‐Value	Cut‐off value^a^	Sensitivity (%)	Specificity (%)	NPV (%)	PPV (%)
PNA‐MFI	0.789 (0.699–0.880)	<0.001	2797	76.2	78.0	83.9	68.8
PT	0.677 (0.560–0.794)	0.002	14.65	63.4	71.2	69.3	61.1
PA (%)	0.621 (0.503–0.739)	0.035	62.14	41.5	94.0	69.3	83.1
INR	0.665 (0.549–0.781)	0.005	1.28	48.8	91.5	71.5	80.3
APTT	0.550 (0.434–0.666)	0.401	28.15	85.4	23.7	69.5	44.3
FIB	0.623 (0.514–0.733)	0.036	343.9	51.2	66.1	65.6	51.8
DD	0.501 (0.376–0.627)	0.986	2.25	36.8	79.0	63.7	55.5
TT	0.596 (0.478–0.714)	0.108	14.95	72.5	37.9	65.9	45.4

Note: PA, prothrombin time activity.

Abbreviations: APTT, activated partial thromboplastin time; INR, international normalized ratio; NPV, negative predictive value; PNA‐MFI, platelet–neutrophil aggregate mean fluorescence intensity; PPV, positive predictive value; PT, prothrombin time; SIC, sepsis‐induced coagulopathy; TT, thrombin time.

^a^All cut‐off values were determined by the Youden index.

### 3.5. High Predictive Value of PNA‐MFI for DIC Among Sepsis Patients

In our study, out of the 101 sepsis patients, 24 developed DIC (DIC group), while 77 did not develop DIC (Non‐DIC group) during their hospitalization term. Supporting Information 5: Table S2 provides comprehensive details regarding the characteristics of the samples. The DIC group showed higher 90‐day mortality, SOFA scores, and incidence of fungal infection compared to the non‐DIC group. Consistent with DIC diagnostic criteria, platelet count in the DIC group was lower than in the non‐DIC group. Additionally, the DIC group exhibited prolonged PT, elevated INR, prolonged thrombin time, reduced PA (%), and decreased FIB compared to the non‐DIC group. All PLAs indicators have been analyzed in both the DIC group and the non‐DIC group. According to the data presented in Supporting Information 6: Table S3, the PNA‐MFI values were substantially greater in the DIC group compared to the non‐DIC group. Similarly, PLyA (T‐lymphocyte)‐MFI values were also higher in the DIC group, as were PLyA (CD8^+^T‐lymphocyte)‐MFI values and PLyA (B‐lymphocyte)‐MFI values. For the prediction of DIC, PNA‐MFI demonstrated significant value, with an AUC of 0.781 (95% CI: 0.680–0.883 and *p*  < 0.001) (Figure 3A and Table 5). Importantly, DeLong‘s test confirmed that its predictive performance was statistically superior to that of several PLA indicators, including PLyA (T‐lymphocyte)‐MFI, PLyA (CD8^+^T‐lymphocyte)‐MFI, and PLyA (B‐lymphocyte)‐MFI (all *p*  < 0.05, by DeLong‘s test) (Table 6). In addition, we observed that D‐dimer does not seem to be a specific indicator for DIC patients in sepsis (Supporting Information 5: Table S2). PNA‐MFI had the highest level of significance in predicting DIC. *p*‐Values for 16 PLA indicators in DIC vs. non‐DIC comparisons were adjusted by FDR correction and Bonferroni correction. Adjusted *p*‐values are presented in Supporting Information 7: Table S4.

Figure 3Association of PLA indicators with sepsis‐induced DIC risk: ROC curves. Figures A, B, C, and D show ROC curves for PNA‐MFI, PLyA (T‐lymphocyte)‐MFI, PLyA (CD8^+^T‐lymphocyte)‐MFI, and PLyA (B‐lymphocyte)‐MFI in predicting DIC, with predictive values represented as AUC. MFI, mean fluorescence intensity; ROC, receiver operating characteristic; PNA‐MFI, platelet–neutrophil aggregate mean fluorescence intensity; PLyA (T‐lymphocyte)‐MFI, platelet T–lymphocyte aggregate mean fluorescence intensity; PLyA (CD8^+^T‐lymphocyte)‐MFI, platelet CD8^+^T–lymphocyte aggregate mean fluorescence intensity; PLyA (B‐lymphocyte)‐MFI, platelet B–lymphocyte aggregate mean fluorescence intensity; DIC, disseminated intravascular coagulation.

**Figure:**
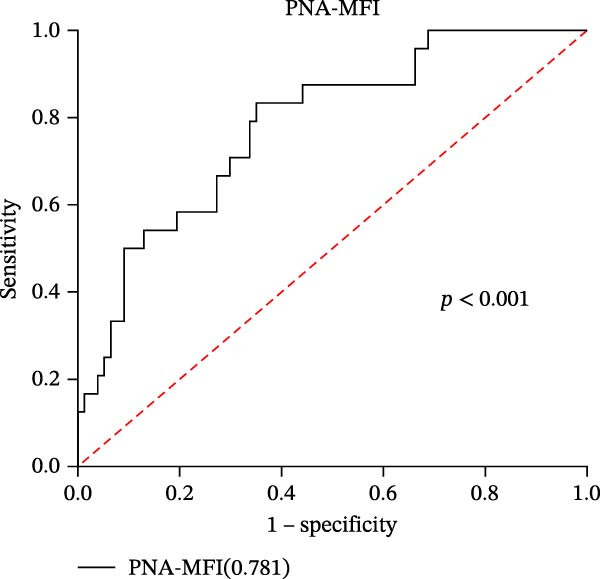
(A)

**Figure:**
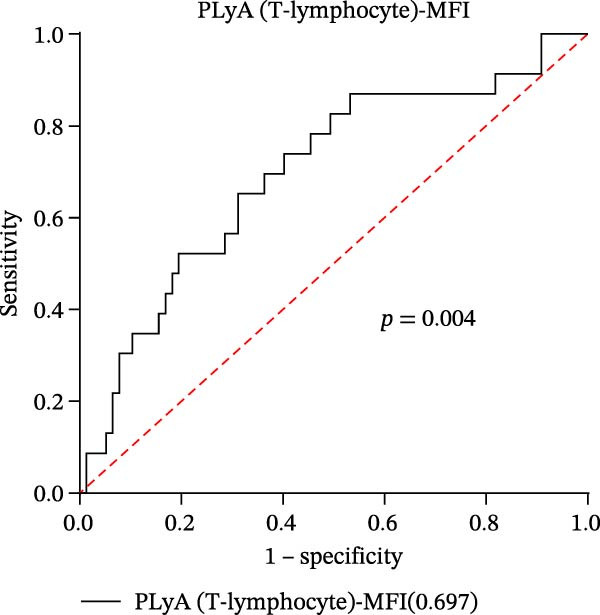
(B)

**Figure:**
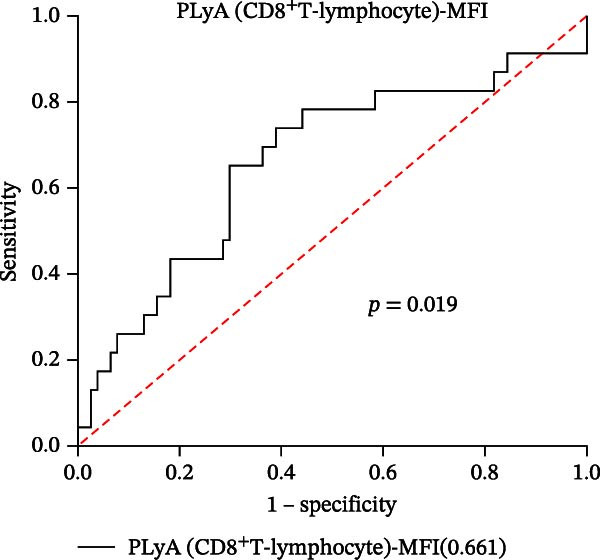
(C)

**Figure:**
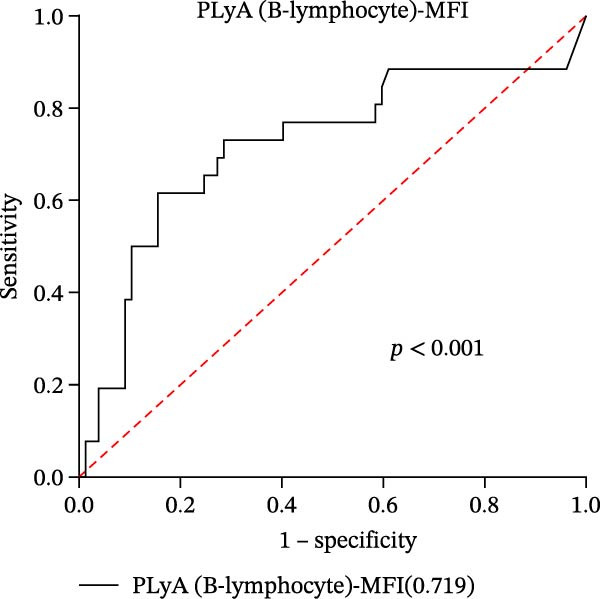
(D)

**Table 5:** Diagnostic test characteristics of PLA indicators for sepsis‐induced DIC.

PLA indicators	AUC (95% CI)	*p*‐Value	Cut‐off value^a^	Sensitivity (%)	Specificity (%)	NPV (%)	PPV (%)
PNA‐MFI	0.781 (0.680–0.883)	<0.001	2621	83.3	64.9	92.6	42.6
PLyA (T‐lymphocytes)‐MFI	0.697 (0.572–0.823)	0.004	2848	73.9	59.7	87.9	36.4
PLyA (CD8^+^T‐lymphocyte)‐MFI	0.661 (0.524–0.797)	0.019	3479	65.2	70.1	86.6	40.5
PLyA (B‐lymphocyte)‐MFI	0.719 (0.591–0.847)	<0.001	3314	73.1	71.4	89.5	44.3

*Note:* PLyA (B‐lymphocyte)‐MFI, platelet B–lymphocyte aggregate mean fluorescence intensity; PLyA (CD8^+^T‐lymphocyte)‐MFI, platelet CD8^+^T–lymphocyte aggregate mean fluorescence intensity; PLyA (T‐lymphocyte)‐MFI, platelet T–lymphocyte aggregate mean fluorescence intensity.

Abbreviations: AUC, area under the curve; MFI, mean fluorescence intensity; NPV, negative predictive value; PNA‐MFI, platelet–neutrophil aggregate mean fluorescence intensity; PPV, positive predictive value.

^a^All cut‐off values were determined by the Youden index.

**Table 6:** Pairwise comparisons of AUCs between PNA‐MFI and other indicators for predicting DIC (DeLong‘s test).

Comparison indicators (vs. PNA‐MFI)	*Z* statistic	*p*‐Value	95% CI for AUC difference
PLyA (T‐lymphocyte)‐MFI	−2.025	0.043	−0.110, −0.002)
PLyA (CD8^+^T‐lymphocyte)‐MFI	−2.049	0.041	(−0.225, −0.005)
PLyA (B‐lymphocyte)‐MFI	−2.076	0.038	(−0.143, −0.004)

*Note: p*‐Value ≤ 0.05 was considered significant. PLyA (B‐lymphocyte)‐MFI, platelet B–lymphocyte aggregate mean fluorescence intensity; PLyA (CD8^+^T‐lymphocyte)‐MFI, platelet CD8^+^T–lymphocyte aggregate mean fluorescence intensity; PLyA (T‐lymphocyte)‐MFI, platelet T–lymphocyte aggregate mean fluorescence intensity.

Abbreviations: CI, confidence interval; MFI, mean fluorescence intensity; PNA‐MFI, platelet–neutrophil aggregate mean fluorescence intensity.

### 3.6. High Predictive Value of PLA Indicators for DIC in Sepsis via K‐M Curves and Log‐Rank Test

To evaluate the predictive significance of PLAs indicators for DIC during hospitalization, we first stratified patients into high‐level and low‐level groups for each identified PLAs indicator using cut‐off values derived from ROC curve analysis (Table 5). Subsequently, Kaplan–Meier (K‐M) curves were constructed to depict the time‐to‐DIC distribution, and log‐rank tests were performed to compare differences between these two groups. Cut‐off values for identified PLAs indicators were determined via ROC curve analysis as follows: PNA‐MFI at 2621, PLyA (T‐lymphocyte)‐MFI at 2848, PLyA (CD8^+^T‐lymphocyte)‐MFI at 3479, and PLyA (B‐lymphocyte)‐MFI at 3314 (Table 5). Septic patients with elevated PNA‐MFI (Figure 4A), PLyA (T‐lymphocyte)‐MFI (Figure 4B), PLyA (CD8^+^T‐lymphocyte)‐MFI (Figure 4C), and PLyA (B‐lymphocyte)‐MFI (Figure 4D) exhibited a higher risk of developing DIC compared to those with lower levels of these markers.

Figure 4Association of PLA indicators with sepsis‐induced DIC risk: Kaplan–Meier curves. Figures A, B, C, and D present Kaplan–Meier curves for PNA‐MFI, PLyA (T‐lymphocyte)‐MFI, PLyA (CD8^+^T‐lymphocyte)‐MFI, and PLyA (B‐lymphocyte)‐MFI in predicting DIC. Patients were divided into two groups according to the cut‐off value. The red line represents the following groups: PNA‐MFI ≥ 2621 (*n* = 54), PLyA (T‐lymphocyte)‐MFI ≥ 2848 (*n* = 52), PLyA (CD8^+^T‐lymphocyte)‐MFI ≥ 3479 (*n* = 62), and PLyA (B‐lymphocyte)‐MFI ≥ 3414 (*n* = 60). The blue line represents PNA‐MFI < 2621 (*n* = 47), PLyA (T‐lymphocyte)‐MFI < 2848 (*n* = 49), PLyA (T‐lymphocyte)‐MFI < 3479 (*n* = 39), and PLyA (B‐lymphocyte)‐MFI < 3414 (*n* = 41). MFI, mean fluorescence intensity; PNA‐MFI, platelet–neutrophil aggregate mean fluorescence intensity; PLyA (T‐lymphocyte)‐MFI, platelet T–lymphocyte aggregate mean fluorescence intensity; PLyA (CD8^+^T‐lymphocyte)‐MFI, platelet CD8^+^T–lymphocyte aggregate mean fluorescence intensity; PLyA (B‐lymphocyte)‐MFI, platelet B–lymphocyte aggregate mean fluorescence intensity; DIC, disseminated intravascular coagulation.

**Figure:**
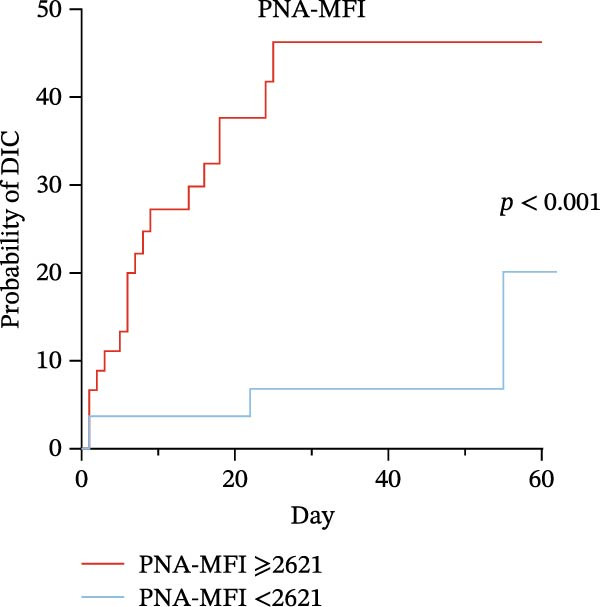
(A)

**Figure:**
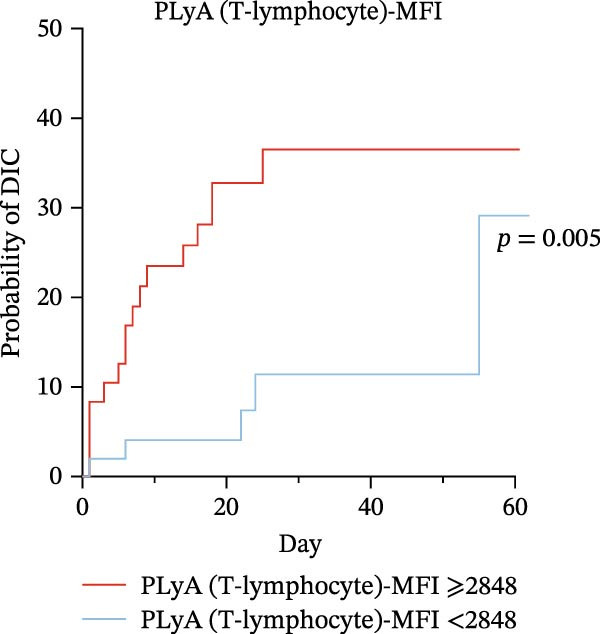
(B)

**Figure:**
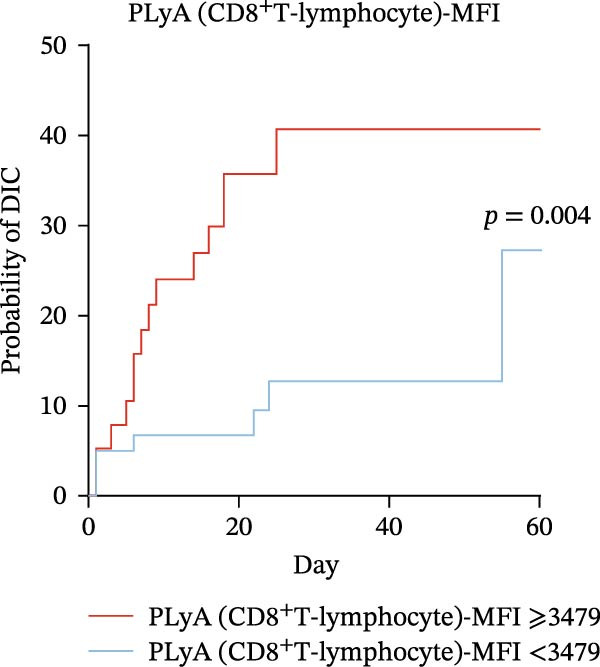
(C)

**Figure:**
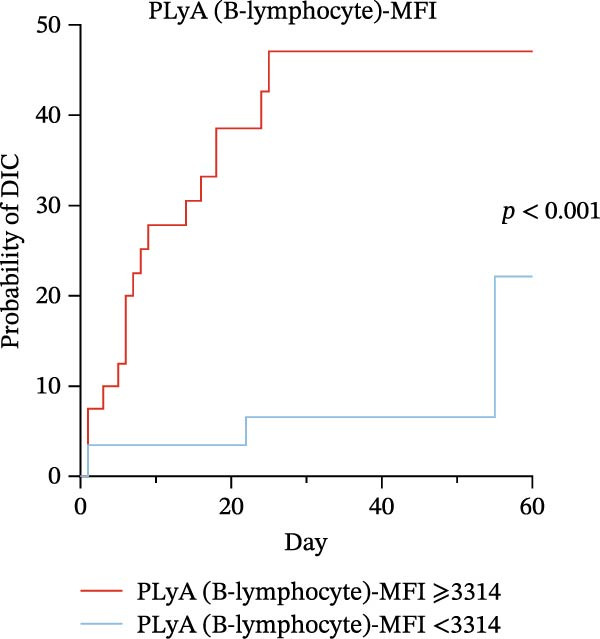
(D)

## 4. Discussion

Our study implicates PNAs in the pathogenesis of SIC and, for the first time, identifies their quantitative measure—PNA‐MFI—as a reliable biomarker for diagnosing SIC and predicting the onset of sepsis‐induced DIC. In summary, our findings suggest that PNA‐MFI serves as a key marker for identifying SIC and forecasting DIC in sepsis patients. Further investigation into the role of PNAs in SIC could clarify its pathogenesis and progression, holding significant clinical relevance.

Sepsis is considered to be related to thrombocytopenia, since infections can trigger platelet activation and aggregation, resulting in decreased platelet count [32]. However, the precise mechanism underlying sepsis‐induced thrombocytopenia remains unclear [33]. For septic patients. we found that platelet count was positively correlated with the percentage of PLAs and inversely correlated with MFI level of PLAs (data not shown). Gawaz et al. [34] suggest a connection between thrombocytopenia and active platelet binding to leukocytes. In a mouse model of sepsis, reduced activated platelet results in a corresponding decrease in platelet–leukocyte complex formation [35]. Critically, our study demonstrates that SIC exhibits distinct PLA dynamics. Whereas general sepsis elevates PLA formation (associated with adverse outcomes) [36, 37], SIC patients paradoxically show reduced PLA formation. We propose these results from severe depletion of activated platelets available for aggregation—a consequence of consumptive coagulopathy.

MFI is a crucial marker in flow cytometric analysis of PLAs, measuring the strength of platelet–leukocyte interactions by detecting platelets on leukocyte surfaces [38]. Elevated MFI in SIC indicates enhanced binding strength of residual platelets to leukocyte surfaces, thereby accelerating platelet clearance. Our study reveals this enhanced platelet–leukocyte interaction in SIC, although the precise underlying mechanism requires further investigation.

Our findings suggest that PNAs may be key contributors to SIC pathogenesis. Platelet–neutrophil interactions play a vital role in inflammation and immune responses [39]. In sepsis, these interactions can promote thrombosis through multiple mechanisms, including chemokines, damage‐associated molecular patterns (DAMPs), high mobility group protein B1 (HMGB1) released by platelets, and myeloperoxidase (MPO) and NETs released by neutrophils [40]. Especially, PNA formation in sepsis can be triggered by LPS via Toll‐like receptor 4 (TLR4) on platelets, which further promotes NET formation [41]. NETs are a critical factor in thrombogenesis, as they capture blood cells and coagulation factors promote platelet aggregation and support the coagulation cascade [42]. Collectively, our study indicates that circulating PNA levels reflect the coagulation status in sepsis, offering new perspectives for the diagnosis and treatment of SIC.

The formation of PLAs may also be influenced by the type of pathogen in septic patients. Due to the complexity of the microbial environment in septic patients—who are rarely affected by a single pathogen—the mechanisms may differ depending on the causative pathogen [43]. In our study, sepsis caused by gram‐positive cocci exhibited higher PEA% and PMA (all)‐MFI levels compared to sepsis caused by gram‐negative bacilli. In viral infections, PMA (classical‐monocyte)% decreases, while in fungal infections, both PLyA (CD4^+^T‐lymphocyte)‐MFI levels and PLyA (CD8^+^T‐lymphocyte)‐MFI levels increase (data not shown). These findings suggest that platelet–monocyte interactions may play a more prominent role in viral sepsis. Furthermore, the role of platelet T‐lymphocyte interactions in fungal sepsis warrants further investigation.

We also explored the relationship between PLA indicators and sepsis severity in septic patients. The results revealed that the percentage levels of aggregates were negatively correlated with disease severity, whereas MFI values were positively correlated (data not shown). This finding is consistent with those reported by Soriano et al. [44].

Flow cytometry is a well‐established technology with proven utility in clinical diagnostics for conditions such as autoimmune diseases and hematological malignancies and is increasingly available in tertiary care centers [45, 46]. Regarding the practical implementation of PNA‐MFI assessment, we acknowledge that flow cytometry—while widely available in tertiary hospitals for platelet function testing—incurs higher operational costs than routine coagulation assays. However, the technique requires ~30 min when performed concurrently with conventional tests (e.g., PT/INR and platelet counts), enabling seamless integration into existing diagnostic workflows without significant time penalties. Furthermore, emerging point‐of‐care cytometers show promise for rapid PNA‐MFI quantification in critical care settings [28]. While incorporating a novel biomarker into established prediction systems like ISTH‐SIC/DIC scores introduces complexity, PNA‐MFI provides unique pathophysiological insights that meaningfully enhance clinical decision‐making. Specifically, it streamlines management in two pivotal scenarios: First, patients with normal SIC scores but elevated PNA‐MFI may benefit from preemptive anticoagulation or intensified monitoring, allowing intervention before meeting conventional coagulopathy criteria. Second, our data demonstrate that low PNA‐MFI levels in SIC patients correlate with minimal DIC risk within 75 days (Figure 4), enabling clinicians to safely reduce unnecessary interventions in this subgroup and reallocate resources to high‐risk individuals. Thus, rather than merely predicting scoring system outcomes, PNA‐MFI quantifies active thromboinflammation to guide precision anticoagulation—transforming reactive treatment into risk‐adapted prevention.

There are certain constraints in our investigation. First, it was a single‐center study with a limited sample size. Additionally, the exploratory pairwise model comparisons were conducted without adjustment for multiple testing. While our results provide preliminary evidence for the association between PLAs and coagulopathy outcomes, they should be considered exploratory hypotheses rather than definitive conclusions—external validation of the PNA‐MFI is indispensable to establish its clinical utility. Larger, multicenter studies are urgently needed to confirm our findings and validate the predictive performance of the PNA‐MFI across diverse populations. Second, further mechanistic research is necessary to elucidate the relationship between platelets and leukocytes. For example, transcriptome sequencing of isolated aggregates could reveal molecular changes during thrombus formation. Finally, we acknowledge a potential limitation regarding our flow cytometry sample preparation. Although our protocol incorporated immediate paraformaldehyde fixation—a well‐established method to stabilize platelets and prevent ex vivo activation—the necessary centrifugation steps could theoretically introduce some artifact [47, 48]. Nevertheless, we emphasize that the uniform processing of all samples ensures that the comparative differences observed between the SIC and non‐SIC groups are robust and reflective of the underlying biology.

## 5. Conclusion

Our research demonstrates a significant correlation between the increase in PNA‐MFI levels and the occurrence of SIC. Moreover, we found that PNA‐MFI exhibited a significant ability to predict the occurrence of DIC. PNA‐MFI serves as a reliable diagnostic indicator for detecting SIC and predicting the onset of DIC in sepsis patients. These findings advance the understanding of PNAs in SIC pathogenesis and introduce a novel tool to aid clinicians in early diagnosis and risk assessment. While our findings demonstrate robust internal validity, external validation in independent, multicenter cohorts is essential prior to clinical translation. Further large‐scale studies are warranted to confirm and generalize these preliminary results across diverse populations.

**Nomenclature**
DIC: Disseminated intravascular coagulation
ICU: Intensive care unit
MFI: Mean fluorescence intensity
PEAs: Platelet–eosinophil aggregates
PGAs: Platelet–granulocyte aggregates
PLAs: Platelet–leukocyte aggregates
PLyAs: Platelet–lymphocyte aggregates
PMAs: Platelet–monocyte aggregates
PNAs: Platelet–neutrophil aggregates
ROC: Receiver operating characteristic
SIC: Sepsis‐induced coagulopathy
SOFA: Sequential organ failure assessment.

## Author Contributions

Tenglong Dai, Shuang Liang, and Jun Wu were involved in the development and planning of the study. Tenglong Dai contributed to the process of data curation. Shuang Liang, Huiru Zhao, and Jun Wu engaged in the formal analysis. Yu Su, Ying Liu, Cuiying Liang, and Xinyang Yue were involved in the project management. Bin Li and Hao Wang had the responsibility of furnishing patient information. Tenglong Dai prepared the manuscript, which underwent thorough evaluation by all writers.

## Funding

This study was supported by the Beijing Municipal Natural Science Foundation (Grants L246006 and L254086), the Beijing Municipal Health Commission High‐Level Public Health Technical Talent Development Project, the Natural Science Foundation Cultivation Project (Grant ZR‐202412) of Beijing Jishuitan Hospital Affiliated to Capital Medical University, and the Wu Jieping Clinical Research Fund.

## Disclosure

The final manuscript for submission has received approval from all authors.

## Ethics Statement

The study protocol was approved by the Ethics Committee of Beijing Jishuitan Hospital, Capital Medical University, May 08, 2024 ([K [2024‐197‐00]).

## Consent

The authors have nothing to report.

## Conflicts of Interest

The authors declare no conflicts of interest.

## Supporting Information

Additional supporting information can be found online in the Supporting Information section.

## Supporting information


**Supporting Information 1** Figure S1: Gating strategy for flow cytometric analysis of platelet–monocyte aggregates, including total monocytes (CD14^+^) and classical monocytes (CD14^++^CD16⁻).

**Supporting Information 2** Figure S2: Gating strategy for flow cytometric analysis of platelet–lymphocyte aggregates, including T‐lymphocytes (CD3^+^), CD4^+^T‐lymphocytes, CD8^+^T‐lymphocytes, and B‐lymphocytes (CD19^+^).

**Supporting Information 3** Figure S3: Gating strategy for flow cytometric analysis of platelet–granulocyte aggregates, including neutrophils (CD11b^+^CD15^+^CD16^+^) and eosinophils (CD11b^+^CD15^+^CD16⁻).

**Supporting Information 4** Table S1: Adjusted *p*‐values for comparisons of PLA indicators between SIC and non‐SIC groups.

**Supporting Information 5** Baseline demographics and laboratory parameters in sepsis patients with and without DIC.

**Supporting Information 6** Table S3: Comparison of PLA indicators between DIC and non‐DIC groups.

**Supporting Information 7** Adjusted *p*‐values for comparisons of PLA indicators between DIC and non‐DIC groups.

## Data Availability

All data generated or analyzed during the present study are included in this published article.
