# Supplementary figures and images for: Circulating Platelet–Neutrophil Aggregates as Novel Biomarker for Coagulopathy Diagnosis and Disseminated Intravascular Coagulation Prediction in Sepsis
Source: Mediators Inflamm. 2026 Mar 23;2026:5580762. doi: 10.1155/mi/5580762 (PMC13140174; doi:10.1155/mi/5580762)

## Slide 1
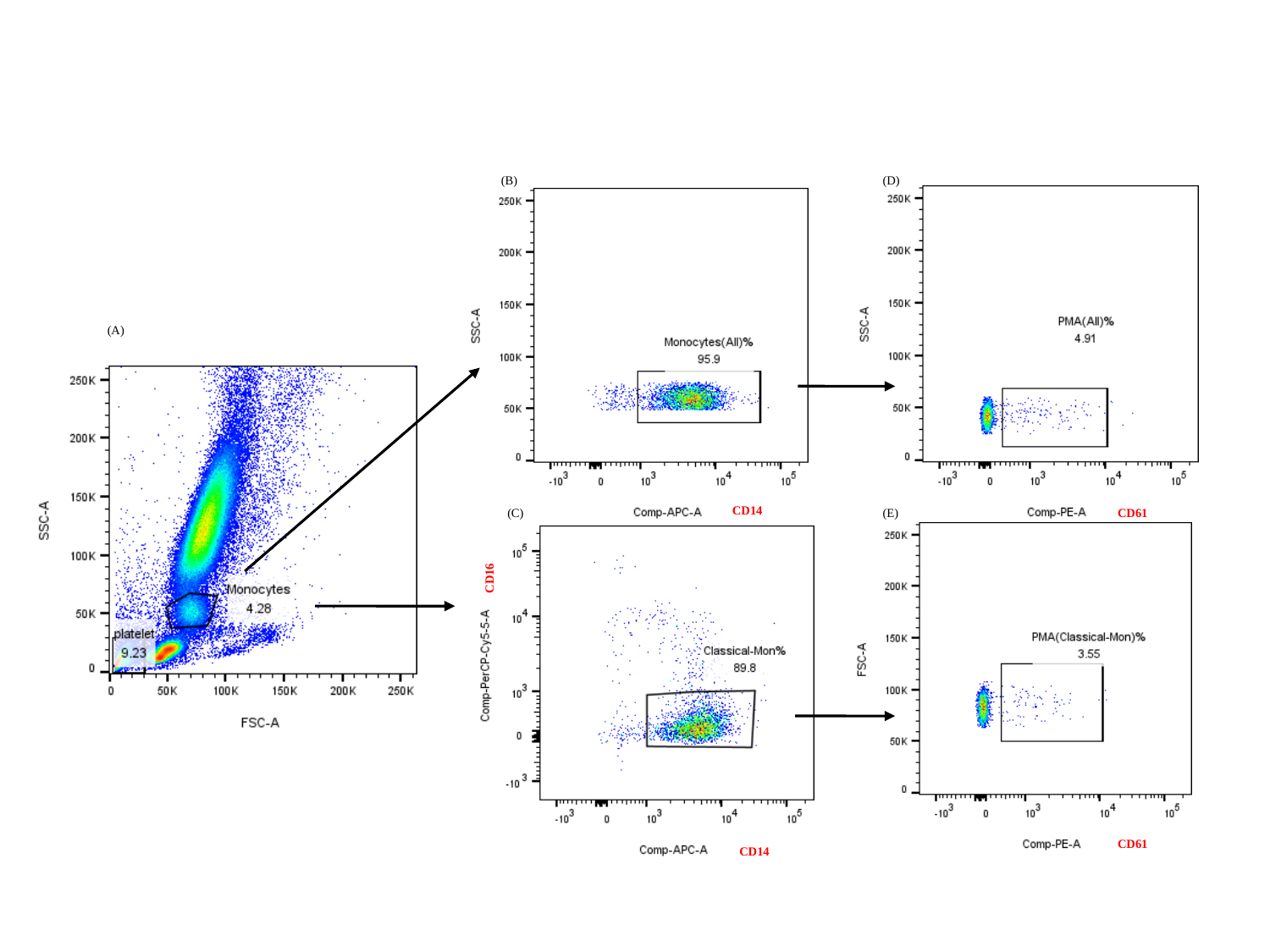

(B)
(D)
(A)
(E)
CD14
CD61
CD16
CD61
CD14
(C)

Supplement: Supplementary file 1 — Supporting Information 1 Figure S1: Gating strategy for flow cytometric analysis of platelet–monocyte aggregates, including total monocytes (CD14+) and classical monocytes (CD14++CD16⁻). [file MI-2026-5580762-s007.ppt]

## Slide 1
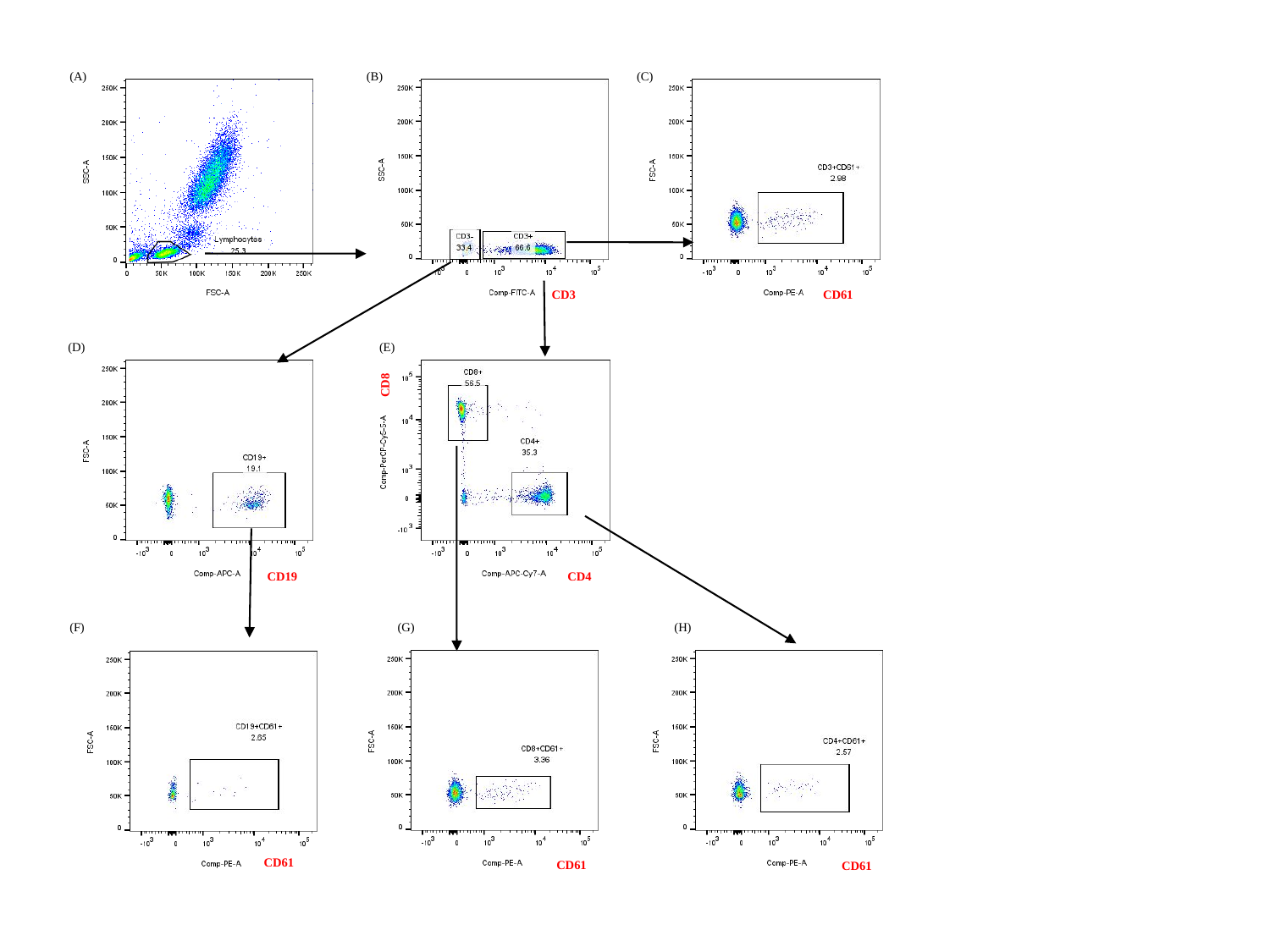

(A)
(C)
(B)
(D)
(E)
(F)
(G)
(H)
CD3
CD61
CD8
CD4
CD19
CD61
CD61
CD61

Supplement: Supplementary file 2 — Supporting Information 2 Figure S2: Gating strategy for flow cytometric analysis of platelet–lymphocyte aggregates, including T‐lymphocytes (CD3+), CD4+T‐lymphocytes, CD8+T‐lymphocytes, and B‐lymphocytes (CD19+). [file MI-2026-5580762-s003.ppt]

## Slide 1
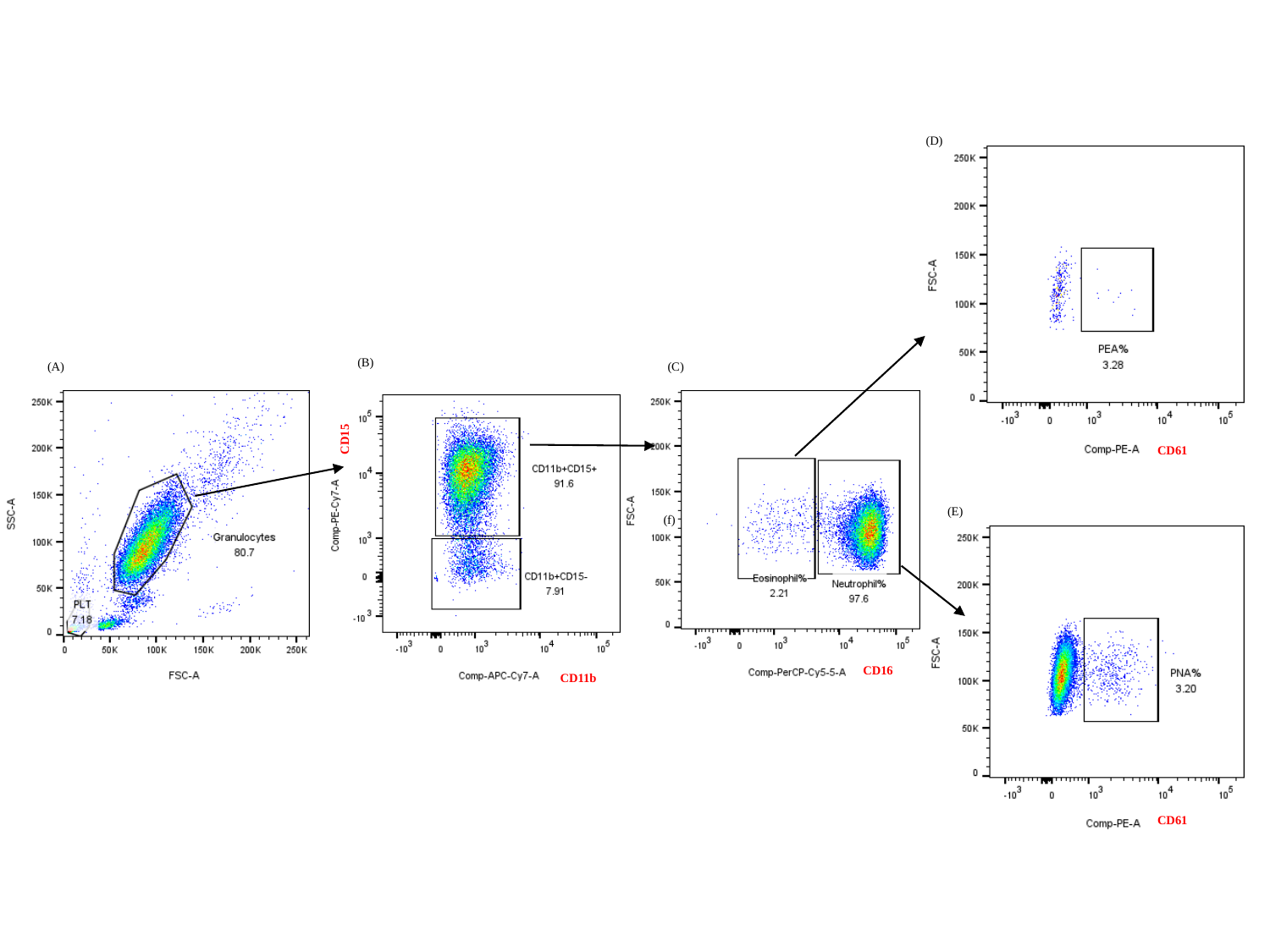

(D)
(B)
(A)
(C)
CD15
CD61
(f)
CD16
CD11b
CD61
(E)

Supplement: Supplementary file 3 — Supporting Information 3 Figure S3: Gating strategy for flow cytometric analysis of platelet–granulocyte aggregates, including neutrophils (CD11b+CD15+CD16+) and eosinophils (CD11b+CD15+CD16⁻). [file MI-2026-5580762-s004.ppt]
