# Supplementary material for: Circulating Platelet–Neutrophil Aggregates as Novel Biomarker for Coagulopathy Diagnosis and Disseminated Intravascular Coagulation Prediction in Sepsis
Source: Mediators Inflamm. 2026 Mar 23;2026:5580762. doi: 10.1155/mi/5580762 (PMC13140174; doi:10.1155/mi/5580762)
Supplement: Supplementary file 4 — Supporting Information 4 Table S1: Adjusted p‐values for comparisons of PLA indicators between SIC and non‐SIC groups. [file MI-2026-5580762-s005.docx]

**Table S1**. **P-Value adjustment for comparing PLA indicators between SIC and non-SIC groups**.

| PLA indicators | | Unadjusted  *P*-value | | FDR-  Adjusted P-value | | | Bonferroni-  Adjusted *P*-value | |
| --- | --- | --- | --- | --- | --- | --- | --- | --- |
| PNA% | | **0.021** | | **0.026** | **0.336** | | |  |
| PEA% | **0.007** | | **0.011** | | | **0.106** | |  |
| PMA (All) % | 0.087 | | 0.099 | | | 1.000 | |  |
| PMA (Classical-monocyte) % | | 0.098 | | 0.102 | 1.000 | | |  |
| PLyA (T-lymphocyte) % | | **0.007** | | **0.011** | **0.109** | | |  |
| PLyA (CD4^+^T-lymphocyte) % | | **0.001** | | **0.003** | **0.022** | | |  |
| PLyA (CD8^+^T-lymphocyte) % | | **0.016** | | **0.022** | **0.261** | | |  |
| PLyA (B-lymphocyte) % | | **0.016** | | **0.022** | **0.261** | | |  |
| PNA-MFI | | **＜0.001** | | **＜0.001** | **0.002** | | |  |
| PEA-MFI | | 0.102 | | 0.102 | 1.000 | | |  |
| PMA (All)-MFI | | **＜0.001** | | **＜0.001** | **0.002** | | |  |
| PMA (Classical-monocyte)-MFI | | **＜0.001** | | **＜0.001** | **0.002** | | |  |
| PLyA (T-lymphocyte)-MFI | | **＜0.001** | | **＜0.001** | **0.002** | | |  |
| PLyA (CD4^+^T-lymphocyte)-MFI | | **＜0.001** | | **0.001** | **0.008** | | |  |
| PLyA (CD8^+^T-lymphocyte)-MFI | | **＜0.001** | | **＜0.001** | **0.002** | | |  |
| PLyA (B-lymphocyte)-MFI | | **＜0.001** | | **＜0.001** | **0.002** | | |  |

P-value≤0.05 was considered significant. FDR, false discovery rate, PNA%, the percentage of platelet-neutrophil aggregates, PEA%, the percentage of platelet-eosinophil aggregates, PMA (All) %, the percentage of platelet-monocyte aggregates, PMA (Classical-monocyte) %, the percentage of platelet Classical-monocyte aggregates, PLyA (T-lymphocyte) %, the percentage of platelet T-lymphocyte aggregates, PLyA (CD4^+^T-lymphocyte) %, the percentage of platelet CD4^+^T-lymphocyte aggregates, PLyA (CD8^+^T-lymphocyte) %, the percentage of platelet CD8^+^T-lymphocyte aggregates, PLyA (B-lymphocyte) %, the percentage of platelet B-lymphocyte aggregates, MFI, mean fluorescence intensity, PNA-MFI, platelet-neutrophil aggregate mean fluorescence intensity, PEA-MFI, platelet- eosinophil aggregate mean fluorescence intensity, PMA (All)-MFI, platelet-monocyte aggregate mean fluorescence intensity, PMA (Classical-monocyte)-MFI, platelet Classical-monocyte aggregate mean fluorescence intensity, PLyA (T-lymphocyte)-MFI, platelet T-lymphocyte aggregate mean fluorescence intensity, PLyA (CD4^+^T-lymphocyte)-MFI, platelet CD4^+^T-lymphocyte aggregate mean fluorescence intensity, PLyA (CD8^+^T-lymphocyte)-MFI, platelet CD8^+^T-lymphocyte aggregate mean fluorescence intensity, PLyA (B-lymphocyte)-MFI, platelet B-lymphocyte aggregate mean fluorescence intensity.
