# Supplementary material for: Circulating Platelet–Neutrophil Aggregates as Novel Biomarker for Coagulopathy Diagnosis and Disseminated Intravascular Coagulation Prediction in Sepsis
Source: Mediators Inflamm. 2026 Mar 23;2026:5580762. doi: 10.1155/mi/5580762 (PMC13140174; doi:10.1155/mi/5580762)
Supplement: Supplementary file 5 — Supporting Information 5 Baseline demographics and laboratory parameters in sepsis patients with and without DIC. [file MI-2026-5580762-s006.docx]

**Table S2**. **Demographics and biomarkers in Sepsis patients with and without DIC***

| Variables | Non-DIC  (N = 77) | DIC  (N = 24) | P value |
| --- | --- | --- | --- |
| Characteristics |  |  |  |
| Male sex, N (%) | 51(67) | 16(67) | 0.862 |
| Age, years | 67.00(57.00,77.00) | 72.00(67.00,77.00) | 0.131 |
| Mortality day 90 N (%) | 10(13) | 13(54) | ＜0.001 |
| Site of infection |  |  | 0.721 |
| Bloodstream infection (Gram positive cocci) | 10(13) | 2(8) |  |
| Bloodstream infection (Gram negative bacilli) | 7(9) | 2(8) |  |
| Multiple infections N (%) | 17(22) | 4(17) |  |
| Pulmonary infection N  (%) | 38(51) | 13(55) |  |
| Unknown N (%) | 4(5) | 3(12) |  |
| Virus N (%) | 10(13) | 7(29) | 0.069 |
| Fungus N (%) | 18(24) | 13(54) | 0.005 |
| Medical conditions |  |  |  |
| Hypertension N (%) | 38(50) | 15(63) | 0.285 |
| Diabetes mellitus N (%) | 21(27) | 9(38) | 0.358 |
| Coronary heart disease N (%) | 18(24) | 7(29) | 0.589 |
| Cerebral infarction N (%) | 16(21) | 3(13) | 0.352 |
| Cancer N (%) | 12(16) | 3(13) | 0.694 |
| Clinical characteristics |  |  |  |
| RBC (10^12^/L) | 2.90(2.55, 3.30) | 2.69(2.48, 2.96) | 0.186 |
| WBC (10^9^/L) | 10.81(7.85,14.76) | 10.22(7.14,12.45) | 0.274 |
| PLT (10^9^/L) | 170(113,256) | 71(56,98) | ＜0.001 |
| PT(s) | 14.20(13.00,15.20) | 15.00(14.10,16.10) | 0.048 |
| PA (%) | 78.60(66.00,89.94) | 69.50(54.30,82.00) | 0.029 |
| INR | 1.15(1.06,1.27) | 1.26(1.12,1.51) | 0.023 |
| APTT(s) | 33.30(28.30,40.21) | 41.50(30.72,49.90) | 0.051 |
| FIB (mg / dl) | 421.00(300.36,548.60) | 329.90(249.67,434.05) | 0.008 |
| D-dimer (mg /l FEU) | 4.18(2.16,7.79) | 5.14(2.98,11.85) | 0.263 |
| TT(s)  SOFA score | 15.50(14.70,17.00)  4.00(2.00, 6.00) | 17.92(15.40,19.10)  6.00(4.00,8.00) | 0.002  ＜0.001 |

*Values are expressed as median (25th, 75th percentiles), or number (percentage). P < 0.05 were considered statistically significant.

PT, prothrombin time, APTT, activated partial thromboplastin time, TT, thrombin time, INR, international normalized ratio, PA, prothrombin time activity, SIC, sepsis-induced coagulopathy, DIC, disseminated intravascular coagulation. SOFA, sequential organ failure assessment.
