# Supplementary material for: Circulating Platelet–Neutrophil Aggregates as Novel Biomarker for Coagulopathy Diagnosis and Disseminated Intravascular Coagulation Prediction in Sepsis
Source: Mediators Inflamm. 2026 Mar 23;2026:5580762. doi: 10.1155/mi/5580762 (PMC13140174; doi:10.1155/mi/5580762)
Supplement: Supplementary file 6 — Supporting Information 6 Table S3: Comparison of PLA indicators between DIC and non‐DIC groups. [file MI-2026-5580762-s001.docx]

**Table S3**. **Distribution difference of PLA indicators in the non-DIC group and the DIC group among septic patients***.

| PLA indicators | Non-DIC  (N = 77) | DIC  (N = 24) | P value |
| --- | --- | --- | --- |
| PNA% | 5.09(3.44, 7.50) | 4.94(2.52, 6.66) | 0.379 |
| PEA% | 5.41(3.01, 10.25) | 4.47(1.96, 10.70) | 0.569 |
| PMA(All)% | 5.62(3.28, 9.30) | 4.31(2.70, 6.71) | 0.099 |
| PMA (Classical-monocyte) % | 5.21(3.05, 8.93) | 3.70(1.83, 6.19) | 0.054 |
| PLyA (T-lymphocyte) % | 6.44(4.04, 10.70) | 6.38(2.30, 9.77) | 0.265 |
| PLyA (CD4^+^T-lymphocyte) % | 5.50(2.85, 7.85) | 3.04(1.51, 10.30) | 0.148 |
| PLyA (CD8^+^T-lymphocyte) % | 7.09(4.18, 11.70) | 6.65(2.56, 12.20) | 0.288 |
| PLyA (B-lymphocyte) % | 7.42(3.87, 11.85) | 7.71(2.76, 13.40) | 0.856 |
| PNA-MFI | 2359(1720, 3256) | 4028(2845, 4617) | ＜0.001 |
| PEA-MFI | 2423(1717, 3373) | 3032(1942, 3783) | 0.175 |
| PMA (All)-MFI | 4173(3024, 6543) | 4964(3853, 8768) | 0.134 |
| PMA (Classical-monocyte)-MFI | 2496(1558, 3741) | 3094(2235, 4271) | 0.169 |
| PLyA (T-lymphocyte)-MFI | 2548(1783, 3695) | 3774(2661, 5235) | 0.004 |
| PLyA (CD4^+^T-lymphocyte)-MFI | 1937(1469, 3117) | 2668(1664, 4016) | 0.258 |
| PLyA (CD8^+^T-lymphocyte)-MFI | 2436(1774, 3832) | 3674(2568, 5278) | 0.019 |
| PLyA (B-lymphocyte)-MFI | 2581(1528, 3429) | 4161(2783, 4731) | ＜0.001 |

The variables were compared using a Mann-Whitney U test. *Values are expressed as median (25th, 75th percentiles). P-value≤0.05 was considered significant. PNA%, the percentage of platelet-neutrophil aggregates, PEA%, the percentage of platelet-eosinophil aggregates, PMA (All) %, the percentage of platelet-monocyte aggregates, PMA (Classical-monocyte) %, the percentage of platelet Classical-monocyte aggregates, PLyA (T-lymphocyte) %, the percentage of platelet T-lymphocyte aggregates, PLyA (CD4^+^T-lymphocyte) %, the percentage of platelet CD4^+^T-lymphocyte aggregates, PLyA (CD8^+^T-lymphocyte) %, the percentage of platelet CD8^+^T-lymphocyte aggregates, PLyA (B-lymphocyte) %, the percentage of platelet B-lymphocyte aggregates, MFI, mean fluorescence intensity, PNA-MFI, platelet-neutrophil aggregate mean fluorescence intensity, PEA-MFI, platelet- eosinophil aggregate mean fluorescence intensity, PMA (All)-MFI, platelet-monocyte aggregate mean fluorescence intensity, PMA (Classical-monocyte)- MFI, platelet Classical-monocyte aggregate mean fluorescence intensity, PLyA (T-lymphocyte)-MFI, platelet T-lymphocyte aggregate mean fluorescence intensity, PLyA (CD4^+^T-lymphocyte)-MFI, platelet CD4^+^T-lymphocyte aggregate mean fluorescence intensity, PLyA (CD8^+^T-lymphocyte)-MFI, platelet CD8^+^T-lymphocyte aggregate mean fluorescence intensity, PLyA (B-lymphocyte)-MFI, platelet B-lymphocyte aggregate mean fluorescence intensity.
