# Supplementary material for: Circulating Platelet–Neutrophil Aggregates as Novel Biomarker for Coagulopathy Diagnosis and Disseminated Intravascular Coagulation Prediction in Sepsis
Source: Mediators Inflamm. 2026 Mar 23;2026:5580762. doi: 10.1155/mi/5580762 (PMC13140174; doi:10.1155/mi/5580762)
Supplement: Supplementary file 7 — Supporting Information 7 Adjusted p‐values for comparisons of PLA indicators between DIC and non‐DIC groups. [file MI-2026-5580762-s002.docx]

**Table S4**. **P-Value adjustment for comparing PLA indicators between DIC and non-DIC groups**.

| PLA indicators | | Unadjusted  *P*-value | | FDR-  Adjusted P-value | | Bonferroni-Adjusted *P*-value |
| --- | --- | --- | --- | --- | --- | --- |
| PNA% | | 0.376 | | 0.430 | | 1.000 |
| PEA% | 0.566 | | 0.604 | | 1.000 | |
| PMA (All) % | 0.098 | | 0.261 | | 1.000 | |
| PMA (Classical-monocyte) % | | 0.054 | | 0.173 | | 0.864 |
| PLyA (T-lymphocyte) % | | 0.262 | | 0.349 | | 1.000 |
| PLyA (CD4^+^T-lymphocyte) % | | 0.147 | | 0.278 | | 1.000 |
| PLyA (CD8^+^T-lymphocyte) % | | 0.285 | | 0.351 | | 1.000 |
| PLyA (B-lymphocyte) % | | 0.854 | | 0.854 | | 1.000 |
| PNA-MFI | | **＜0.001** | | **0.002** | | **0.002** |
| PEA-MFI | | 0.174 | | 0.278 | | 1.000 |
| PMA (All)-MFI | | 0.133 | | 0.278 | | 1.000 |
| PMA (Classical-monocyte)-MFI | | 0.168 | | 0.278 | | 1.000 |
| PLyA (T-lymphocyte)-MFI | | **0.004** | | **0.022** | | **0.067** |
| PLyA (CD4^+^T-lymphocyte)-MFI | | 0.255 | | 0.349 | | 1.000 |
| PLyA (CD8^+^T-lymphocyte)-MFI | | **0.020** | | **0.080** | | **0.320** |
| PLyA (B-lymphocyte)-MFI | | **＜0.001** | | **0.007** | | **0.014** |

P-value≤0.05 was considered significant. FDR, false discovery rate, PNA%, the percentage of platelet-neutrophil aggregates, PEA%, the percentage of platelet-eosinophil aggregates, PMA (All) %, the percentage of platelet-monocyte aggregates, PMA (Classical-monocyte) %, the percentage of platelet Classical-monocyte aggregates, PLyA (T-lymphocyte) %, the percentage of platelet T-lymphocyte aggregates, PLyA (CD4^+^T-lymphocyte) %, the percentage of platelet CD4^+^T-lymphocyte aggregates, PLyA (CD8^+^T-lymphocyte) %, the percentage of platelet CD8^+^T-lymphocyte aggregates, PLyA (B-lymphocyte) %, the percentage of platelet B-lymphocyte aggregates, MFI, mean fluorescence intensity, PNA-MFI, platelet-neutrophil aggregate mean fluorescence intensity, PEA-MFI, platelet- eosinophil aggregate mean fluorescence intensity, PMA (All)-MFI, platelet-monocyte aggregate mean fluorescence intensity, PMA (Classical-monocyte)-MFI, platelet Classical-monocyte aggregate mean fluorescence intensity, PLyA (T-lymphocyte)-MFI, platelet T-lymphocyte aggregate mean fluorescence intensity, PLyA (CD4^+^T-lymphocyte)-MFI, platelet CD4^+^T-lymphocyte aggregate mean fluorescence intensity, PLyA (CD8^+^T-lymphocyte)-MFI, platelet CD8^+^T-lymphocyte aggregate mean fluorescence intensity, PLyA (B-lymphocyte)-MFI, platelet B-lymphocyte aggregate mean fluorescence intensity.
